# Robust Representation and Nonlinear Spectral Integration of Harmonic Stacks in Layer 4 of the Mouse Primary Auditory Cortex

**DOI:** 10.1523/ENEURO.0038-26.2026

**Published:** 2026-03-18

**Authors:** Yunru Chen (陈韵如), Chih-Ting Chen (陳峙廷), Yuhan Gui (桂语含), Patrick O. Kanold

**Affiliations:** ^1^Department of Biomedical Engineering, Johns Hopkins University School of Medicine, Baltimore, Maryland 21205; ^2^ Kavli NDI, Johns Hopkins University School of Medicine, Baltimore, Maryland 21205; ^3^ Department of Neuroscience, Johns Hopkins University School of Medicine, Baltimore, Maryland 21205; ^4^ Center for Hearing and Balance, Johns Hopkins University, Baltimore, Maryland 21205

**Keywords:** auditory processing, functional connectivity, harmonic stacks, in vivo imaging, spectral integration

## Abstract

Harmonicity is a property of complex sounds such as vocalizations or music, but it remains unclear how harmonicity is processed in the auditory cortex (ACtx). Subregions of ACtx are thought to process harmonic stimuli differently. Selective responses to sound features in ACtx emerge hierarchically from primary ACtx (A1) L4 and secondary ACtx (A2) layer (L)2/3, which is believed to be the most responsive to harmonic sounds. Since harmonic stacks can range from 2 to >10 components, being more similar to naturalistic vocalizations, harmonic sensitivity might also arise hierarchically across layers and areas. We studied responses to harmonic stacks of 2–10 frequencies across A1 L4, A1 L2/3, and A2 L2/3 in adult male and female mice using in vivo two-photon microscopy. We found harmonic-sensitive neurons (HNs) responding only to harmonic stacks but not to individual frequencies in all areas at similar proportions. HNs showed highly nonlinear spectral integration of harmonic frequencies that decreased as the harmonic stacks became more complex. Specifically, onset-biased HNs showed greater nonlinearity than offset-biased HNs only in A1 L4. Moreover, HNs in A1 L4 exhibited higher signal correlation than A2 L2/3. Sound-responsive neurons in A1 L4 have the weakest noise correlation compared with A1 L2/3 and A2 L2/3. Together, harmonic sensitivity is not a unique feature of A2 L2/3 but is already established in A1 L4, where neurons robustly encode harmonic sounds through sparse connections.

## Significance Statement

Harmonics are essential in auditory perception, influencing how we process complex sounds like music and speech. This study reveals that neurons in the cortical layer (L)4 and L2/3 integrate simple and complex harmonic structures with distinct mechanisms of neuronal recruitment. A1 L4 harmonic-sensitive neurons (HNs) demonstrated strong responses through high signal correlation and weak noise correlation, suggesting a robust but sparse mechanism for spectral integration. Our results show that harmonic relationships are already extracted at the input layers of A1 and that HNs show nonlinear facilitative integration. Thus, tuning to sounds of complex spectral contents might be a fundamental processing function of the auditory cortex and is already established in A1 L4, which receives major thalamic inputs.

## Introduction

Harmonicity is a fundamental feature of vocalizations that is perceptually important in humans and mice (Ehret and Riecke, 2002). Harmonic stacks are compound sounds of one or more frequencies that have a positive integer multiple of the fundamental frequency (*F*_0_; Terhardt, 1974; Plack, 2010; McLachlan et al., 2013). As a central hub for sound processing, the auditory cortex (ACtx) encodes the spectral and temporal information of sounds to form a neural representation (Zatorre and Belin, 2001; Kadia and Wang, 2003; Linden et al., 2003; Bendor et al., 2012; Herdener et al., 2013) through sparse coding (Hromadka et al., 2008; Liang et al., 2019; Kang and Kanold, 2024). Specifically, neurons with selective response to harmonic stacks exist in ACtx of ferrets (Bizley et al., 2010), cats (Abeles and Goldstein, 1970; Phillips and Irvine, 1981; Sutter and Schreiner, 1991), and marmosets (Kadia and Wang, 2003; Bendor and Wang, 2005; Song et al., 2016). Such neurons are believed to perform nonlinear summation to integrate multiple frequencies to encode a harmonic sound (Kline et al., 2021).

In mice, behavioral studies reveal that a high proportion of low-frequency harmonic vocalizations are associated with stress (Grimsley et al., 2013, 2016) and serve as a distress call to listeners (Chen et al., 2009). It is critical to understand how ACtx encodes the spectrotemporal information in such harmonic sounds. In mouse A1, layer (L)2/3 and L4 display distinct responses of pure tones (PTs) and unique integration patterns for the spectral content of harmonic stacks (Sołyga and Barkat, 2022). Others suggested that A2 L2/3 is preferentially responsive to harmonics versus PTs compared with A1 and anterior auditory field (Kline et al., 2021). Thus, harmonic processing might vary between ACtx subareas and layers. As L4 neurons receive primary thalamic inputs (Winer et al., 2005; Sherman et al., 2013; Ji et al., 2016) and L2/3 neurons receive inputs from both L4 and other L2/3 neurons within auditory cortical subregions through horizontal connections (Oviedo et al., 2010; Covic and Sherman, 2011; Watkins et al., 2014; Meng et al., 2017; Chang and Kanold, 2021), harmonicity might be processed differentially by specialized neuronal populations distributed across layers in primary and higher-order auditory fields. Additionally, marmoset A1 neurons show selective responses to more complex harmonic structures (Song et al., 2016; Feng and Wang, 2017) suggesting that neurons may integrate simpler and more complex harmonic stacks differently. Moreover, neurons do not work in isolation; thus, it remains unclear whether a fundamental neural network recruits additional neurons or strengthens neuronal connections to process more complex harmonics stacks (i.e., 10 frequencies with *F*_0_ = 4 kHz) that are composed of simpler harmonic stacks (i.e., two frequencies with *F*_0_ = 4 kHz).

In this study, we aimed to determine (1) whether and how harmonics of varying spectral complexity recruit neurons across A1 L4, A1 L2/3, and A2 L2/3 and (2) whether the encoding of harmonic stacks differs among the three populations in A1 L4, A1 L2/3, and A2 L2/3. We imaged the sound-evoked activity in the left ACtx of awake adult CBAxThy1GCaMP6s F1 mice with good high-frequency hearing (Frisina et al., 2011; Bowen et al., 2020) using in vivo two-photon Ca^2+^ imaging. We find that harmonic-sensitive neurons (HNs) are present and distributed in similar proportions across A1 L4, A1 L2/3, and A2 L2/3. HNs integrate component frequencies nonlinearly, but integration became more linear with increasing harmonic complexity. Distinctly, coactivated HNs in A1 L4 showed higher signal correlations but weaker noise correlations than in A2 L2/3, suggesting more independent synaptic inputs but robust encoding of harmonic stacks. Our findings indicate that harmonic processing is not a unique feature of L2/3 but already exists in A1 L4, providing a consistent, sparse, and robust representation of harmonic stimuli across a varied number of components. Such representation supports subsequent processing of harmonic sounds in upper cortical layers, underscoring harmonic encoding as a core process in cortical sound computation.

## Materials and Methods

### Animal preparation

For in vivo two-photon imaging, mice were 12–24 weeks old at the time of the experiments. Mice (*n* = 28) of both sexes were first generation (F1) of CBA/CaJ (Jax #000654) and C57BL/6J-Tg(Thy1-GCaMP6s)GP4.3Dkim/J (Jax #024275) crosses. For electrophysiology recording and the in vivo two-photon imaging experiments used in the analysis of frequency response area (FRA) with three levels of sound amplitude, mice were on a C57BL/6J background with fixed-hearing (cdh23^Ahl/ahl^; Babola et al., 2025). All mice were housed with a reverse light cycle (12 h light, 12 h darkness) and all experiments were conducted during the dark cycle of the mice. All animal procedures were approved by the Johns Hopkins University Animal Care and Use Committee.

### Surgery

In brief, mice were prepared for surgery by inducing anesthesia with 4% isoflurane in 100% oxygen and later reduced to 1–2% isoflurane for maintenance until the surgery ended. Dexamethasone (2 mg/ml) was injected 1 h before the surgery started to prevent inflammation. During the surgery, body temperature was maintained with a feedback-controlled heating pad maintained at 35–36°C. Hair on the top of the head was shaved and removed using a hair remover (Nair) followed by disinfections with 70% ethanol and iodine. The skin and soft tissues were then exposed by detaching and pushing away muscle on the surface of the skull. A craniotomy of a circular area with 4 mm diameter was performed above the left ACtx, covering both A1 and A2, using a dental drill. A stack of round glass coverslips (one 4 mm glass, catalog #640724-CS4R, Warner Instruments, on two stacked 3 mm glasses, catalog #64-0720-CS-3R, Warner Instruments, and fixed with optic glue, catalog #NOA71, Norland Products) was secured onto the exposed brain with SuperGlue around the edge of the window. The exposed skull was covered with dental cement (C&B Metabond). To prepare mice for imaging, a customized metal headplate was fixed onto the cement along the midline of the skull. Carprofen (5 mg/kg) and cefazolin (500 mg/kg) were injected subcutaneously after the surgery and in the following 1–2 d. Mice rested in the home cage and recovered for at least 14 d before the first imaging session. Electrophysiology: The surgical craniotomy procedure was similar to the above procedures, except that the dura was removed. The exposed brain was covered by a 4 mm glass with saline filling the gap. We used silicone adhesive (KWIK-SIL, World Precision Instruments) to seal the cranial window for the ease of removal before the experiment. Cefazolin, dexamethasone, and meloxicam (5 mg/ml) were injected after surgery, and the animal was put in a recovery cage for at least 1 h before the experiment started.

### Sound stimuli

**Figure 1.**
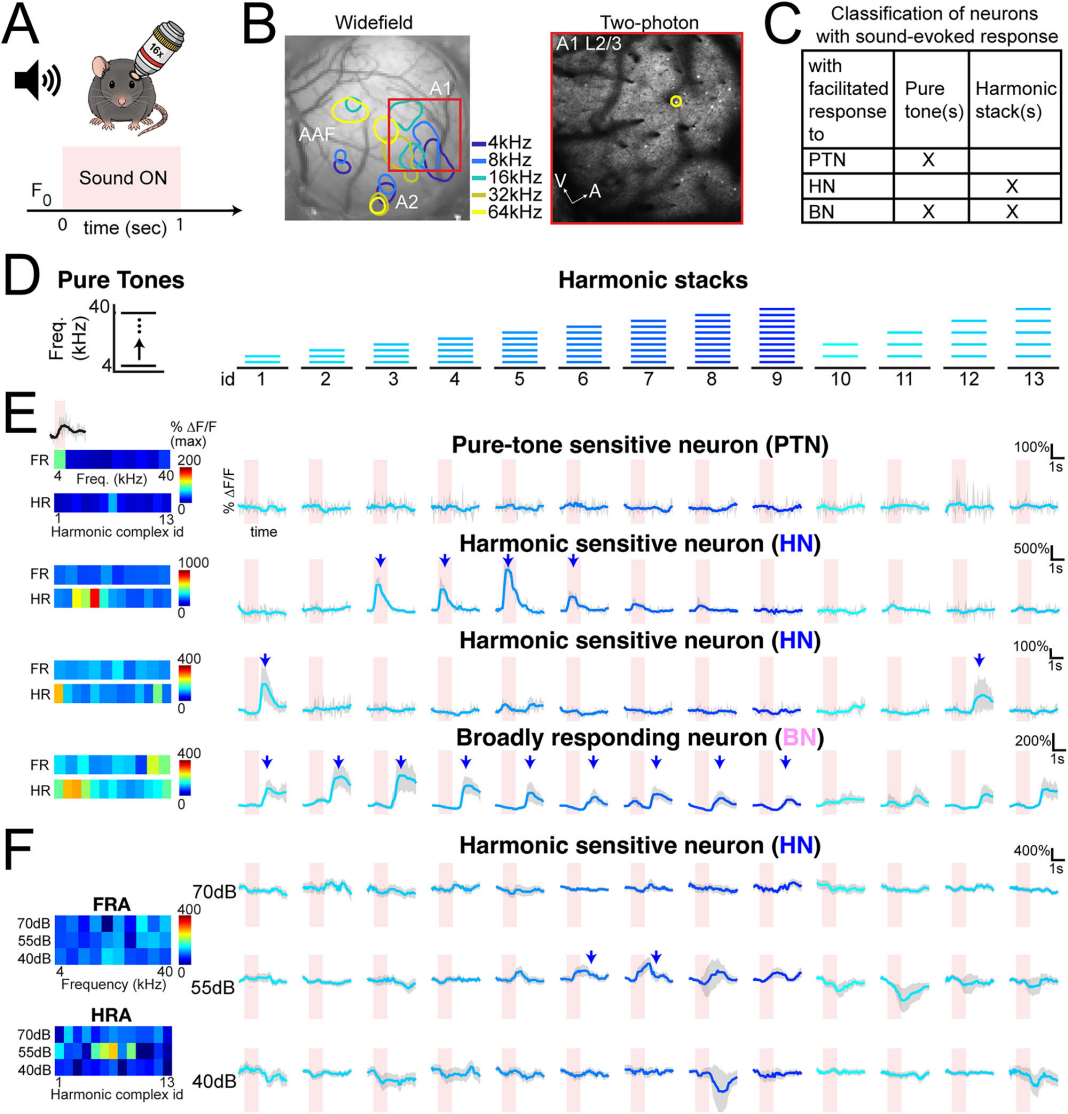
Excitatory neurons in A1 L4, A1 L2/3, and A2 L2/3 are found sensitive to harmonic stacks but not to individual frequencies. ***A***, In vivo two-photon imaging schematic (top) and sound stimulus trial (bottom). *F*_0_, baseline of fluorescence changes in the window of neuron response to the sound stimulus. The pink-shaded area indicates when sound is present. ***B***, Left, Tonotopic map (A1, A2, AAF) identified by widefield imaging of sound-evoked areas. Right, Example field of view of in vivo two-photon calcium imaging of A1 L2/3 excitatory neurons. ***C***, A decision chart of classifying neurons into three categories based on their sound-evoked–facilitative responses to PTs and/or harmonic stacks. ***D***, Schematic of stimulus design. PT, or single frequency, is shown in black. Harmonic stacks consisting of more than one frequency components are shown in blue. Harmonic stacks of id 1–9 has *F*_0_ = 4 kHz. Harmonic stacks of id 10–13 has *F*_0_ = 8 kHz. ***E***, Examples of fluorescence changes compared with baseline (Δ*F*/*F*) of neurons responding to only PTs (PTN), only to the harmonic stacks (HN) with onset or offset response, or both harmonic(s) and PT(s) (BN; from top to bottom). For all example neurons, their average responses to the harmonic stacks are shown as blue-colored traces with gray-shaded standard error of the mean (SEM) across 10 trials of each unique stimulus. The pink-shaded areas denote when sound is present. The maximum Δ*F*/*F* of the average response to PTs (FR) as well as harmonic stacks (HR) are plotted as color blocks on the left column. Gray-shaded area underneath the average traces denotes SEM. ***F***, Example of one HN responding to PTs and harmonic stacks played at three sound levels, 70 dB (top row), 55 dB (middle row), and 40 dB (bottom row). Arrows points out detected significant facilitated response to the corresponding stimulus. Maximum Δ*F*/*F* of the average response to PTs at three sound amplitudes (FRA) as well as harmonic stacks at three sound amplitudes (HRA) are plotted on the left column.

All sound stimuli were pregenerated using MATLAB (MathWorks, version R2023a). Stimuli were loaded into a Tucker-Davis Technologies (TDT) RX6 processor and presented by a ES1 speaker, via a PA5 attenuator, 10 cm away from the right ear of the mouse. The speaker was first calibrated using customized MATLAB (MathWorks, version R2020b) scripts by recording 4–64 kHz PT sounds at 70 dB sound pressure level (SPL), with a calibrated microphone to find the transfer function of the speaker. We then calculated the inverse of this function, which, when added to the speaker transfer function, equalizes the speaker's output. We ensured that the recorded sound level of each frequency component was <1 dB from the target for all sounds played using MATLAB (MathWorks, version R2023a). To study the effect of the complexity of the spectral contents in a harmonic complex, we generated harmonic complexes that are composed of 2–10 frequencies with *F*_0_ = 4 kHz as well as complexes that are composed of two to five frequencies with *F*_0_ = 8 kHz. Similar to multiple published studies that focus on the encoding of complex sounds in mice (Wang et al., 2020; Kline et al., 2021; Sołyga and Barkat, 2022; Kang and Kanold, 2024; Efron et al., 2025), we chose this frequency range because sounds composed of low-to-mid frequencies have been shown in mouse vocalizations, pup calls, and alarm calls in mice, which makes the encoding of these sounds relevant and potentially critical to social behaviors (Ehret and Bernecker, 1986; Ehret and Riecke, 2002; Grimsley et al., 2013, 2016). To generate harmonic stacks with varied spectral complexities, each frequency component was generated at 60 dB SPL and stacked to generate the harmonic stacks without further attenuation. By doing this, each frequency component during presentations of harmonic stacks or PTs has the same sound intensity. The sound intensity of resulting harmonic stacks can vary from 63 dB (2-tone harmonic) to 70 dB (10-tone harmonic). By playing each frequency component in the stack at the same sound amplitude as it is played as PT, we controlled for the input drive to the recorded neurons. This is not achieved by playing both harmonic stacks and PT at exactly the same sound levels, as the individual components of harmonic stacks might fall below the neuron's intensity threshold. For spectrally shifted two-tone harmonic stacks (SH), we generated each frequency component similarly and stacked two 60 dB SPL frequency components, so the sound intensity of the resulting two-tone SH was 63 dB SPL, comparable to two-tone harmonic stacks. During two-photon imaging, each session started with a 10 s of silence period to record the spontaneous activity of neurons. Each trial comprises of 0.5 s of prestimulus silence, 1 s of sound presentation, followed by 3.5 s poststimulus silence (Fig. 1*A*). The order of sound presentation, including harmonic stacks and PTs, is pseudorandomized to ensure all stimuli are played once before beginning the next randomized sequence. Each sound was repeated for 10 times.

### In vivo two-photon calcium imaging

The location of the ACtx and subareas, including A1, A2, and AAF, was determined based on their characteristic tonotopic axes using widefield imaging (Liu et al., 2019). During widefield imaging, mice were head-fixed on a customized imaging station under a 2× objective. Mice were presented with 100 ms 4–64 kHz PT frequencies in three sound levels (60, 75, and 90 dB SPL). Mice were head-fixed on a customized imaging station and imaged with a two-photon microscope (Bruker) using a 16× objective (Nikon) within a sound-attenuating chamber. In one imaging session, harmonic stacks and the stacks individual frequency components were randomized and played as described above. GCaMP6s was excited at 940 nm (Coherent Discovery), and the field of view contained 1,024 × 1,024 pixels covering 1,120 × 1,120 μm^2^. Images were acquired from either L2/3 (150–230 μm below the surface) or L4 (370–430 μm below the surface) using the Prairie View software at 15 fps. Data from each subarea were acquired in separate sessions. For imaging in A2 L2/3, we used a 1.1–1.5× zoom to restrict the field of view to contain only the tonotopy-mapped A2. Sound stimulation was synchronized with the image acquisition using a hardware trigger signal.

### Two-photon data analysis

Motion correction and cell extraction were performed using Suite2P with denoising (Pachitariu et al., 2017) yielding neuron and neuropil fluorescence traces. Pixels overlapping more than one cell were excluded from processing. Neuron fluorescence was neuropil corrected as follows: *F*_cell, corrected_ = *F*_cell_ − 0.9 × *F*_neuropil_. We calculated the change of fluorescence (Δ*F*/*F*) during the response period by dividing fluorescence 3 s after the sound onset from each trial by the average fluorescence of silent frames in 0.5 s preceding the sound onset (*F*_0_). To determine that a neuron has facilitated response to a sound, we calculated the confidence for *F*_0_ and Δ*F*/*F* in all trials of one sound, respectively, and set our criterion to be that the lower bound of Δ*F*/*F* must be larger than the upper bound of *F*_0_ at 5% confidence level. The suppressed response is not considered in this study.

### FRA analysis

To obtain the FRA, we played the PT stimuli at three sound amplitudes, 40, 55, and 70 dB SPL, respectively, to the hearing-fixed mice in C57Bl/6J background (Babola et al., 2025). For harmonic stimuli, we calibrated individual frequency to the desired sound amplitude and generated the harmonic stacks composed of the amplitude-calibrated frequencies. The classification of FRA shape is similar to our previous work (Liu and Kanold, 2021).

### Classification of neurons by sound-evoked response

To categorize neurons into harmonic-, PT-, or both-sensitive neurons, we compared their sound-evoked response calculated as described above. Neurons with no significant facilitative response to any PT but with significant response to any harmonic stacks were categorized as HNs. Similarly, those with no significant facilitative response to any harmonic stacks but with significant facilitative response to any PT sounds were categorized as PT-sensitive neurons (PTNs). Those with significant facilitative response to both harmonic stacks and PT sounds were considered as both-sensitive neurons (BNs).

### *K*-means clustering

To identify distinct types of response, we employed the *K*-means clustering analysis. The average response of sound-responsive neurons to individual sounds was first standardized using *Z*-score normalization. The optimal number of clusters was determined using the elbow method, which identified the turning point of within-cluster sum of squares to be the number of clusters. We then performed the *K*-means clustering to the normalized response by using the MATLAB function “*k*means” with a maximum of 100 iterations.

### Linearity analysis

To evaluate the linearity and nonlinearity of harmonic neurons responding to harmonic stacks, we used customized MATLAB scripts to perform univariate and multivariate linear regressions as well as support vector regression (SVR) for nonlinear regression on the mean response to PT frequencies and harmonic stacks. For univariate linear regression, we used the MATLAB function “polyfit” with degree of 1 to linearly reconstruct harmonic response, *y_n_*, by adjusting the coefficient assigned to each PT response *X* separately. *n* represents reconstructed harmonic response by response to PT *n*. We reported the highest *R*^2^ values of all univariate regressions for one harmonic response:

$$y_{n}=a_{n}X_{n}.$$

For multivariate linear regression, we used MATLAB function “regress” to perform linear reconstruction by response to all corresponding PT components:

$$y=\overset{N}{\underset{n=1}{\sum }}\;a_{n}X_{n}.$$

To evaluate the effects of varied number of predictors on the multivariate regression, we permuted the predictor data or response to PT by shuffling the time frames of the mean response, then performed multivariate regression again, and compared the *R*^2^ between original data and permuted data. To perform SVR analysis, we used the MATLAB function “fitrsvm” with the radial basis function (“rbs”) kernel and data standardization.

### Granger causality analysis

We investigated the functional connectivity (FC) of imaged neurons by performing Granger causality (GC) analysis on the Δ*F*/*F* traces of HNs and BNs. We used the multivariate GC framework implemented in the MVGC toolbox (Barnett and Seth, 2014). For neuron in this analysis, we extracted all trials for all sounds and concatenate all traces into one time series data, which were then detrended and *z*-scored. To reduce the effect of slow fluctuations, we applied a first-order difference filter to each time series data. GC was then estimated using vector autoregressive models fit to each pair of neuron traces between every HN and every BN within each FOV. The optimal model order was selected to be 1 due to the slow calcium dynamics.

Statistical significance of GC links between one HN and any BN within the same FOV was determined by comparison to a nonparametric null distribution generated through bootstrapped surrogate data. For each BN–HN pair, we generated five surrogate traces by permutating frames of the BN's time series data, totally disrupting the temporal dependency between the pair. GC values from real data were considered significant if they exceeded the 95th percentile of the surrogate distribution (*p* < 0.05, one-tailed). To obtain ΔGC values, we subtracted the real GC value of each BN–HN pair by the mean of GC values of surrogate data.

All GC values were calculated across the entire stimulus period (or spontaneous window, where relevant), and results were analyzed at the individual neuron-pair level and aggregated to quantify the distribution of ΔGC strength across sound conditions and subareas. Only neuron pairs with sufficient activity (above threshold signal variance and number of time points) were included in the final analysis. The distance between BN and HN is normalized by dividing the absolute distance by the maximum value of distances of all BN–HN pairs within the same FOV. To perform Spearman correlation analysis, we used the built-in MATLAB function “corr” with the type of correlation being “Spearman.”

### Onset–offset bias index (OBI)

We investigated the OBI using the same methods described in our previous study (Liu et al., 2019). Neurons included in this analysis are sampled from nonoverlapping cortical areas, imaged in different sessions and animals. For each neuron, we defined the onset response window to be the 0.5 s immediately following sound onset and the preonset baseline window to be 0.5 s before the sound onset. Similarly, we defined the offset response window to be 0.5 s immediately following the sound offset and the baseline for the offset response to be 0.5 s before the sound offset. For each neuron, we first averaged the Δ*F*/*F* of 10 trials for each window and then calculated the onset response as the mean activity in the onset window minus the mean of the preonset window and the offset response as the mean activity in the offset window minus the mean of the preoffset window. These values were then used to compute the OBI as follows:

OBI=(offsetresponse–onsetresponse)/(offsetresponse+onsetresponse).

### Electrophysiology experiment and data preprocessing

**Figure 2.**
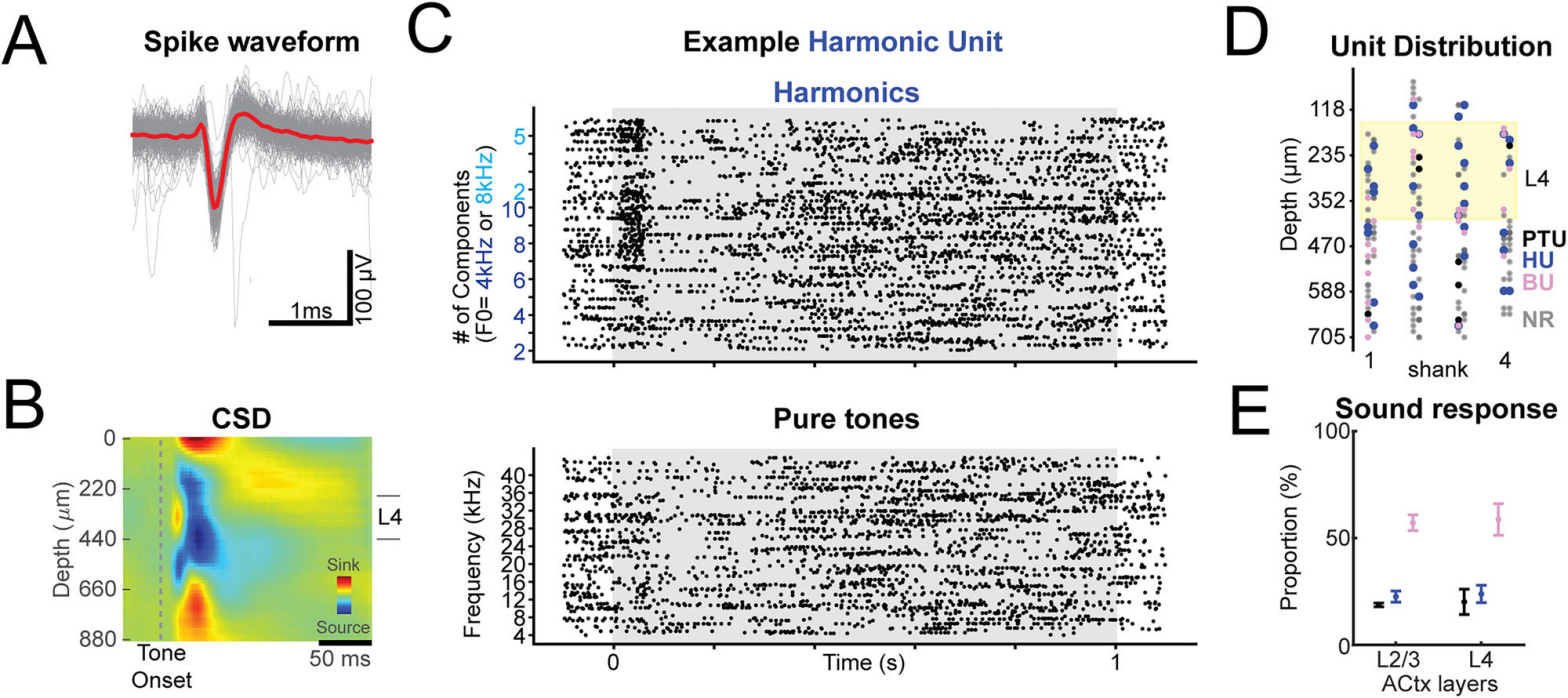
Electrophysiology recording confirms existence of HNs in ACtx. ***A***, Example waveform of spikes from one identified unit cluster in electrophysiological recordings. Gray lines show the 200 example traces aligned based on spikes. The red line shows the average of the 200 aligned traces. ***B***, Example of CSD analysis. L4 is identified by short-latency current sink after sound onset. L2/3 shows a current source and is located above L4. Depths were measured from first electrode that showed an LFP. ***C***, Example raster plots of a recorded unit that showed increased spike counts within 0.2 s window after the sound onsets of harmonic stacks (bottom) and nonsignificantly changed spike counts after tone onset (top). The gray area indicates the presence of sound (1 s). ***D***, Distribution of depth location units in a representative recording on each shank of the four-shank Neuropixel array. Sound-responsive units are classified as HN (blue), PTN (black), or BN (purple) using the same criteria as in vivo two-photon imaging. Units that have no significant sound-evoked response are labeled gray. The yellow-shaded area indicates L4. HN units can be found in L4. ***E***, Quantification of average proportions of responding units (5 animals, 433 sound-responsive units) in L2/3 and L4 of ACtx.

Extracellular recordings were performed with a four-shank high–density probe (Neuropixels 2.0; Steinmetz et al., 2021) in an anechoic chamber, where the animal was head-fixed and awake during the recording. Audio stimuli were presented with a free-field speaker (ES1, TDT) positioned 10 cm from the animal's right ear, contralateral to the recording hemisphere. The speaker was driven by a preamplifier (ED1, TDT) receiving input from a data acquisition device (NI USB-6343). Neuropixel recordings were acquired using the SpikeGLX software system, and the data were digitized at 30 kHz. The neural recording system and the sound stimulation were coordinated by custom scripts in MATLAB. For spike analysis, a global demuxed CAR (common average referencing) filter and a bandpass filter (300–9,000 Hz) were applied to correct for temporal misalignment across channels due to multiplexing during analog-to-digital conversion at the electrode sites and remove irrelevant signals using the postprocessing tool CatGT. Spike sorting was done by Kilosort4 (Pachitariu et al., 2023), and we only included those clusters that were classified as single units in the analysis. We used current source density (CSD) analysis to localize the relative laminar position of each recording site. The CSD was derived as the second derivative of the local field potential (LFP; Mitzdorf and Singer, 1978) with a spacing of 200 μm. The LFPs were obtained from applying a bandpass filter (0.1–500 Hz) to the recordings of all channels. L4 was characterized by a short-latency sink after the stimulus onset, whereas the peaks of current sources marked L2/3 and L5. For quantification of neuron proportion responding to harmonic stacks only, PT only, or both, we imaged three mice with sound duration as 1,000 ms and interstimulus intervals as 1,000–1,500 ms. In addition, we imaged two mice with sound duration as 50 ms and interstimulus interval as 200–250 ms which is more consistent with parameters used in electrophysiology studies. As we found HNs in both groups with different sound parameters, we merged two groups for the analysis of quantification of neuron classes described in Figure 2*E*.

### Analysis of electrophysiology data

To determine if a single unit was responsive to the stimuli, we counted the spike number before and after the stimulus onset for each trial and used paired *t* test to determine whether the spike count within 200 ms after the sound onset is significantly different from the spike count within 200 ms before the sound onset, when the duration of sound is 1,000 ms. Similarly, to account for the offset response, we used a paired *t* test to determine whether the spike count within 200 ms before the sound offset is significantly different (*p* < 0.001) from the spike count within 200 ms after the sound offset. For the experiments conducted with 50 ms duration of sound, the window for spike counts is set to 0.1 s to compare the spike counts before and after sound onset for onset response, as well as before and after sound offset for offset response. Clusters with significant onset or offset response with increased spike counts were then further classified into three categories: PTN, HN, and BN based on whether the clusters had evoked facilitated response to PTs and/or harmonic stacks.

### Correlation analysis

To compute signal correlations, the pair-wise cross-correlation of sound-evoked activity was calculated between cotuned neurons using 2 s after the sound onset, covering 1 s sound onset window as well as the offset response window (1 s after offset), similar to our previously published studies (Winkowski and Kanold, 2013). The same window is used for calculating noise calculation. To compute the pair-wise noise correlations between each pair of sound-responsive neurons, Δ*F*/*F* of each trial of a sound is subtracted by the mean Δ*F*/*F* of all trials of the sound, and then the cross-correlation between the time sequences was calculated for each trial between paired neurons with significant sound-evoked facilitated response by using the MATLAB function “xcorr” at zero lag and normalized by using “coeff” as the normalization parameter.

### Statistical analysis

Statistical tests were performed in MATLAB R2024b unless otherwise indicated. The Lilliefors test was used to test for normal distribution. For data that do not form normal distribution, a two-sided Wilcoxon rank-sum test followed by Bonferroni’s correction for multiple comparisons or a Kruskal–Wallis test followed by Dunn–Sidak for multiple-comparison correction was performed. For data that follow normal distribution, analysis of variance (ANOVA) was performed followed by post hoc *t* test and corrected by Tukey–Kramer. Comparisons of metrics with individual data points, being neuron pairs or individual neurons, were conducted in MATLAB R2025b by applying the linear mixed-effect model to account for the nonindependence of data points collected from the same animal, followed by ANOVA or Kruskal–Wallis depending on the normality of the data.

## Results

To explore the neural representation of harmonic stacks across three auditory subregions (A1 L4, A1 L2/3, and A2 L2/3), we first used widefield imaging to identify tonotopic maps and localize A1 and A2 in each mouse (Fig. 1*B*) as described previously (Liu et al., 2019). We then performed in vivo two-photon imaging in A1 L4, A1 L2/3, and A2 L2/3 in separate sessions (three sessions per animal) on awake young adult mice (A1 L4, *n* = 11 animals; A1 L2/3, *n* = 10 animals; A2 L2/3, *n* = 10 animals). We used F1 mice with a CBA background, which retain high-frequency hearing into adulthood (Frisina et al., 2011;  Bowen et al., 2020), to ensure responses to the high sound frequencies in complex harmonic stacks. We presented either PTs or harmonic stacks with varied numbers of harmonic frequencies (2–10 frequencies, each frequency calibrated at 60 dB during PT and H conditions), covering from 4 to 40 kHz at 60–70 dB SPL with fundamental frequencies (*F*_0_) of 4 or 8 kHz (Fig. 1*C*).

### HNs exhibit high sensitivity for harmonic tuning at one sound level

The ACtx is thought to have a sparse representation of sound features, so we first investigated whether a subset of excitatory neurons responded exclusively to harmonic stimuli, without responding to any PT components of the harmonic stacks, and whether different ACtx subfields displayed varying fractions of harmonically responsive neurons. We generated harmonic stacks in which each component frequency is calibrated at 60 dB, so the composed sound is played at 63–70 dB (2-tone to 10-tone). We recorded sound-evoked responses to harmonic stimuli with varied component numbers, as well as to individual PTs (A1 L4, 10 animals, 4,974 neurons; A1 L2/3, 12 animals, 10,913 neurons; A2 L2/3, 7 animals, 1,792 neurons). Imaging sessions were conducted in A1 L4, A1 L2/3, and A2 L2/3 when the targeted subarea was clearly identified in widefield imaging and was suitable for two-photon imaging. We classified neurons into three types based on their facilitated (positive DF/F) responses to sounds, in which every frequency is tuned at 60 dB: PTNs (responsive only to PTs), HNs (responsive only to harmonic stacks), and BNs (responsive to both PTs and harmonic stacks; Fig. 1*D*).

The responses of ACtx neurons can vary with the sound level, and therefore ACtx neurons can have diverse FRA (Liu and Kanold, 2021). To identify the proportion of BNs and PTN neurons in each FRA type, we investigated the FRA of neurons in A1 L2/3 by imaging the responses to PTs and harmonic stacks at three sound levels (40 dB, 55 dB, and 70 dB; *n* = 3 mice, 1,889 sound-responsive neurons). For harmonic stacks, each frequency component was calibrated to 40, 55, or 70 dB and then combined to generate the harmonic stacks. We were still able to identify PT, harmonic-responsive neurons that respond exclusively to PTs or harmonic stacks, as well as both-responsive neurons. By characterizing the FRA of PT-responsive neurons (i.e., PTNs and BNs), we recovered six distinct shapes of FRA that were previously identified in our previous work (Liu and Kanold, 2021; Maximov and Kanold, 2025). For each neuron, we calculated the best frequency, which elicited the highest response, and used the best frequency as the center of the FRA. We then clustered the centered FRA of each as in our previous work (Liu and Kanold, 2021), into one of the clusters V, H, I, S1, S2, S3 (Extended Data Fig. 1-1). We found that the tSNE plot exhibited distinct clusters (Extended Data Fig. 1-1*A*). BN and PTN occupied different regions of the tSNE plots (Extended Data Fig. 1-1*B*) which are further analyzed by quantifying the proportions of BN and PTN in each cluster (Extended Data Fig. 1-1*C*). We found that the proportions of PTNs in S1 and S3 clusters are significantly higher than the proportions of BNs (Extended Data Fig. 1-1*C*). This result further suggests that, compared with PTNs, BNs tend to have broader tuning reflected by FRA shape, and they respond to more neighboring frequencies around the best frequencies when the sound becomes louder.

10.1523/ENEURO.0038-26.2026.f1-1 Figure 1-1 **Distinct frequency response area (FRA) profiles of both-responding neurons (BNs) and pure-tone neurons (PTNs) in A1 L2/3.** **A**: t-SNE visualization of k-means clustering applied to centered FRA profiles from sound-responsive neurons (n = 3 animals, 1418 neurons). **B**: Same t-SNE plot as in (A), now colored by BN and PTN identity, illustrating the distribution of neuron subtypes across FRA-based clusters. **C**: Proportion of BNs and PTNs within each cluster, showing differential cluster occupancy by neuron subtype. Error bars indicates standard errors. *: *p* < 0.05. Two-sample t-test on proportions of BN versus PTN for each cluster: V, *p* *=* 0.4125; I, *p* *=* 0.4543; H, *p* *=* 0.1531; S1, *p* *=* 0.0445; S2, *p* *=* 0.0680; S3, *p* *=* 0.0249. **D**: Average centered FRA maps for each of the six clusters, revealing distinct spectral and intensity tuning profiles. Vertical bars indicate peak response frequency. Download Figure 1-1, TIF file.

Together with our criteria for classifying neurons as BNs in Figure 1, these results further suggest that BNs may contribute more to the encoding of spectral combinations and may serve as an intermediate population bridging basic and complex spectral features, whereas PTNs provide precise frequency resolution at a given sound level.

### Electrophysiology recording of HN response to harmonic stacks in both L4 and L2/3

Two-photon imaging in deeper layers has the potential of contamination by neuropil signal from more superficial layers. Thus, to confirm our observation of HNs in L4 by two-photon imaging, we performed electrophysiological recordings in awake, hearing-fixed mice (Babola et al., 2025). To record across the depth of A1, we used Neuropixel 2.0 arrays (Steinmetz et al., 2021) and identified cortical layers using CSD analysis (Fig. 2*B*). We identified recording locations in L4 as sites showing the first current sink shortly after sound onset as L4 and sites 200 µm above the early current sink as L2/3. We identified single units with significant sound response within 0.2 after onset or offset and found units that show similar sensitivity to harmonic sounds (HNs) but not to PTs (Fig. 2*C*). We found HNs in both A1 L2/3 and L4 (Fig. 2*D*). This result is consistent with our in vivo two-photon imaging results (Fig. 1) and suggests the existence of neurons that are responsive selectively to harmonic stacks but not to any individual PTs in both L4 and L2/3 of A1.

### Harmonic sensitivity is prevalent in multiple ACtx subareas

**Figure 3.**
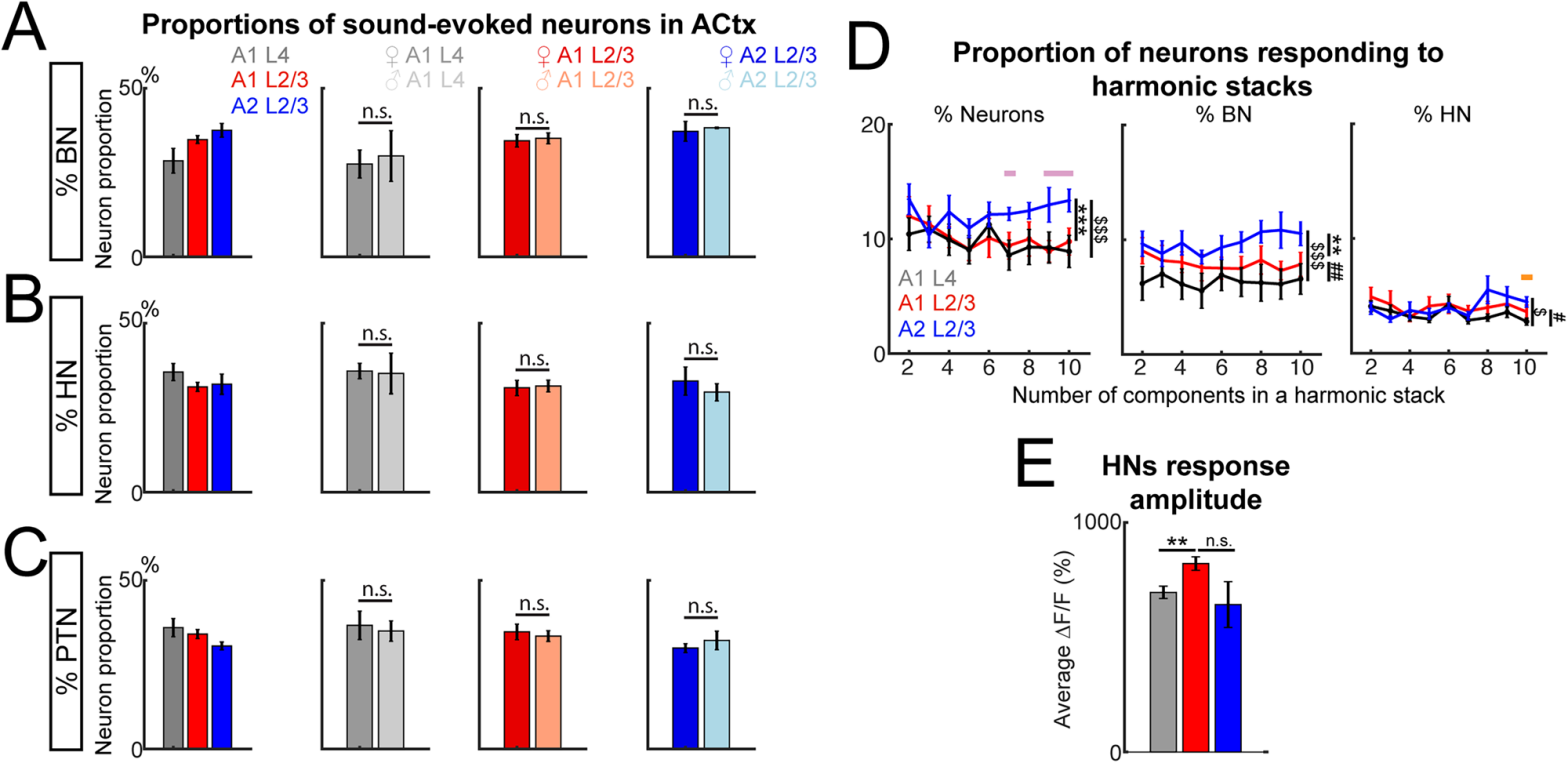
Harmonic neurons are similarly sparse in three imaged auditory subareas. ***A***, Left, Average proportions of BNs in A1 L4 (gray), A1 L2/3 (red), and A2 L2/3 (blue). Middle and right, Comparisons of BN proportions in females and males in three subareas. A1 L4, female, 27.54% ± 10.17%; male, 29.92% ± 15.05%. One-way ANOVA on the effect of sex, *F*_(1, 8)_ = 0.0907; *p* = 0.77. A1 L2/3, female, 34.44% ± 4.56%; male, 35.17% ± 3.96%. One-way ANOVA on the effect of sex, *F*_(1, 10)_ = 0.0877; *p* = 0.7732. A2 L2/3, female, 37.22% ± 6.6%; male, 38.26% ± 0.34%. One-way ANOVA on the effect of sex, *F*_(1, 5)_ = 0.0443; *p* = 0.8416. Error bars indicate SEM. ***B***, Similar to ***A*** but average proportions of HNs. A1 L4, female, 35.81% ± 5.62%; male, 35.12% ± 11.97%. One-way ANOVA on the effect of sex, *F*_(1, 8)_ = 0.0157; *p* = 0.9035. A1 L2/3, female, 30.86% ± 5.44%; male, 31.37% ± 4.3%. One-way ANOVA on the effect of sex, *F*_(1, 10)_ = 0.0326; *p* = 0.8604. A2 L2/3, female, 32.89% ± 9.22%; male, 29.58% ± 3.54%. One-way ANOVA on the effect of sex, *F*_(1, 5)_ = 0.2211; *p* = 0.6580. ***C***, Similar to ***A*** but average proportions of PTNs. A1 L4, female, 36.64% ± 10.31%; male, 34.96% ± 6.06%. One-way ANOVA on the effect of sex, *F*_(1, 8)_ = 0.0850; *p* = 0.7780. A1 L2/3, female, 34.70% ± 5.68%; male, 33.46% ± 3.93%. One-way ANOVA on the effect of sex, *F*_(1, 10)_ = 0.1907; *p* = 0.6692. A2 L2/3, female, 29.89% ± 3.01%; male, 32.15% ± 3.88%. One-way ANOVA on the effect of sex, *F*_(1, 5)_ = 0.7131; *p* = 0.4369. ***D***, Proportions of neurons responding to harmonic stacks with varied number of harmonic frequencies in a stack in A1 L4 (left), A1 L2/3 (middle), and A2 L2/3 (right; Extended Data Fig. 3-1). All data are represented as mean ± SEM. The number of symbols indicate significance with post hoc test on subareas (Extended Data Fig. 3-2). *p < 0.05; **p < 0.01; ***p < 0.001. * denotes comparison between A1 L2/3 and A2 L2/3. # denotes comparison between A1 L2/3 and A1 L4. $ denotes comparison between A1 L4 and A2 L2/3. Colored horizontal bars (pink, A1 L2/3 vs A2 L2/3; orange, A2 L2/3 vs A1 L4) indicates significant difference (*p* < 0.05) by paired *t* test with Bonferroni’s corrections among subareas for marked sound conditions. ***E***, Response amplitude of HNs responding to their characteristic harmonic sound. Error bars indicate mean ± SEM. A1 L4, 6.95 ± 0.27; A1 L2/3, 8.21 ± 0.29; A2 L2/3, 6.42 ± 1.00. Wilcoxon rank-sum test with Bonferroni-corrected *p* values, A1 L4 versus A1 L2/3, *p* = 0.012; A1 L2/3 versus A2 L2/3, *p* = 0.36; A1 L4 versus A2 L2/3, *p* = 0.056.

We next quantified the recruitment of neurons to encode sounds in A1 L4, A1 L2/3, and A2 L2/3 by calculating the proportion of PTNs, HNs, and BNs responding to PTs and harmonic stacks played at one sound level. All three subregions exhibited similar proportions of HNs (Fig. 3*B*; A1 L4, 35.54% ± 8.09%; A1 L2/3, 31.12% ± 4.68%; A2 L2/3, 31.94% ± 7.83%), PTNs (Fig. 3*C*; A1 L4, 35.97% ± 8.49%; A1 L2/3, 34.08% ± 4.70%; A2 L2/3, 30.54% ± 3.12%), and BNs (Fig. 3*A*; A1 L4, 28.50% ± 11.60%; A1 L2/3, 34.80% ± 4.09%; A2 L2/3, 37.52% ± 5.42%). One-way ANOVA revealed no significant effect of the subarea factor on the proportions of HNs (*F*_(2, 26)_ = 1.24; *p* = 0.307), PTNs (*F*_(2, 26)_ = 1.67; *p* = 0.21), nor BNs (*F*_(2, 26)_ = 3.17; *p* = 0.059), suggesting comparable sparseness in responsiveness to the presented PTs and harmonic stacks across these areas. Male and female mice had similar proportions of HNs (Fig. 3*B*), PTNs (Fig. 3*C*), and BNs (Fig. 3*A*) in all three subareas. As the currently examined metrics did not reveal sex-specific differences, data from both sexes were combined for further analyses.

10.1523/ENEURO.0038-26.2026.f3-1 Figure 3-1 **Statistics of HN proportions activated by varied harmonic stacks** Report of statistics of average HN proportion across animals for each subarea and each harmonic sound. Download Figure 3-1, DOCX file.

10.1523/ENEURO.0038-26.2026.f3-2 Figure 3-2 **Two-sample t-test comparing HN proportions between subareas for each sound** Statistical report of pair-wise comparisons across HN proportions activated by harmonic sounds with different numbers of frequencies, following two-way ANOVA on main factors of subareas and number of harmonic frequencies. Download Figure 3-2, DOCX file.

We further investigated whether more neurons are recruited to encode a harmonic stack of more frequencies, as one neuron responding to a five-tone harmonic stack might also be responsive to a six-tone stack with one harmonic frequency added to the original five-stack. However, we found that the proportion of responsive neurons within subareas remained largely consistent as the harmonic stacks became more complex (Fig. 3*D*; Extended Data Fig. 3-1). A two-way ANOVA examined two main factors of subareas and number of harmonic frequencies, revealing the significant effect of subareas but not number of harmonic frequencies on the proportions of HNs (subareas, *F*_(2, 234)_ = 11.5; *p* = 1.72 × 10^−5^; number of frequencies, *F*_(8, 234)_ = 1; *p* = 0.43; interactions between subareas and component numbers, *F*_(16, 234)_ = 0.61; *p* = 0.87). Specifically, A2 L2/3 shows a higher proportion of neurons encoding harmonic stacks, regardless of the number of component frequencies, compared with A1 L2/3 and A1 L4 (post hoc test A1 L4 vs A1 L2/3, *p* = 0.75; A1 L2/3 vs A2 L2/3, *p* = 1.67 × 10^−4^; A1 L4 vs A2 L2/3, *p* = 1.84 × 10^−5^; Fig. 3*D*, left; Extended Data Fig. 3-2). When splitting the proportion of harmonic-responding population into BNs and HNs, we found that A2 L2/3 still showed a larger fraction of BNs than A1 L4 and L2/3 across a range of number of frequencies (two-way ANOVA, subareas, *F*_(2, 234)_ = 19.52; *p* = 1.44 × 10^−8^; component numbers, *F*_(8, 234)_ = 0.31; *p* = 0.96; interactions, *F*_(16,234)_ = 0.31; *p* = 0.99; post hoc, A1 L4 vs A1 L2/3, *p* = 0.0028; A1 L2/3 vs A2 L2/3, *p* = 0.0014; A1 L4 vs A2 L2/3, *p* = 1.33 × 10^−9^; Fig. 3*D*, middle). A2 L2/3 also showed a significantly larger proportion of HNs responding to harmonic stacks than A1 L4 but not A1 L2/3 (subareas, *F*_(2, 234)_ = 4.99; *p* = 0.0076; component numbers, *F*_(8, 234)_ = 1.97; *p* = 0.051; interactions, *F*_(16, 234)_ = 1.21; *p* = 0.26; post hoc, A1 L4 vs A1 L2/3, *p* = 0.013; A1 L2/3 vs A2 L2/3, *p* = 0.99; A1 L4 vs A2 L2/3, *p* = 0.029; Fig. 3*D*, right). These results indicated that all subareas encode harmonic sounds sparsely, with little summation of response for more complex harmonic stacks. Moreover, our results also indicate that A2 L2/3 contains a larger proportion of neurons responding to harmonic stacks, especially BNs.

The proportion of activated neurons reflects the network size that represents harmonic stacks, while the response amplitudes of individual neurons may reflect the robustness of the neuronal network's sound representation, which is crucial for stable perception. We thus calculated the response amplitude of each HN to its “best harmonic”—the harmonic stimulus eliciting the strongest average response. Our results revealed that HNs in A1 L2/3 showed significantly higher DF/F to their best harmonic stacks compared with HNs in A1 L4 (Fig. 3*E*). Together, these findings suggest that neurons in all three subregions exhibit sensitive tuning to harmonic stacks, with HNs in A1 L2/3 showing the highest response to harmonic stacks. Additionally, our results showed that the sensitivity is already observed at the thalamorecipient layer (A1 L4), suggesting that the source of harmonic responsiveness could reflect both cortical processing and thalamic inputs. However, the higher response amplitudes in A1 L2/3 suggest a more robust representation of the harmonic stacks in this layer compared with A1 L4. We observed no differences in amplitude between A2 L2/3 and the other two subareas, suggesting that A2 L2/3 HNs are more heterogeneous in their response amplitudes to the same sounds, which are represented significantly differently in A1 L2/3 and A1 L4.

**Figure 4.**
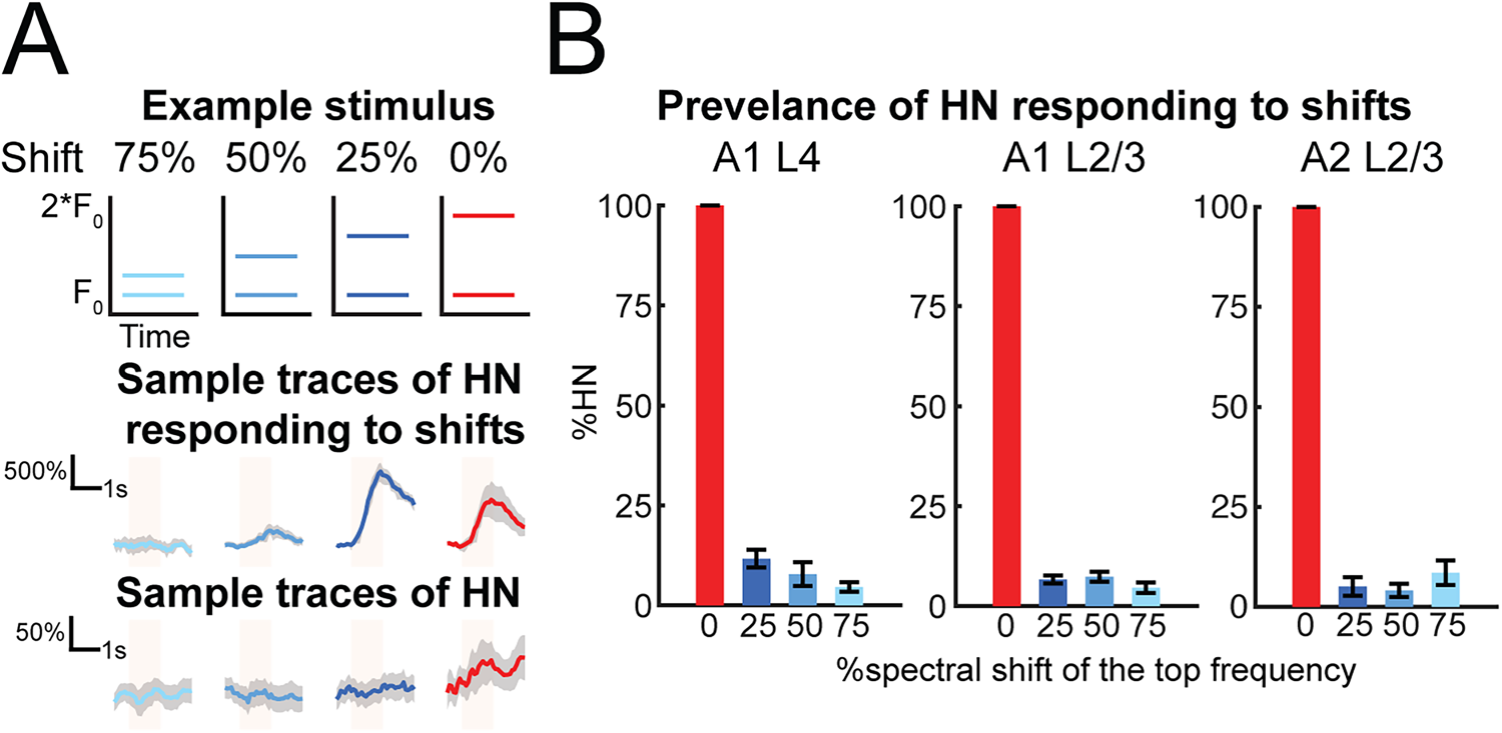
Most of HNs are sensitive to frequency shifts in harmonic stacks and faithfully respond to the harmonic stacks. ***A***, Example of one two-tone harmonic and its spectrally shifted stacks (top) and example of Δ*F*/*F* of neurons only responding to the harmonic (blue) but not spectral shifts (orange), as well as shift-tolerant harmonic neurons (SHN) with facilitated response to both the harmonic and its spectral shifted versions but not to any PT frequencies that makes up the composite sounds. ***B***, Small fractions of HN populations, identified as SHN, in A1 L4 (25% shift, 11.65% ± 2.22%; 50% shift, 7.77% ± 2.95%; 75% shift, 4.56% ± 1.19%), A1 L2/3 (25% shift, 11.65% ± 0.81%; 50% shift, 6.52% ± 1.31%; 75% shift, 5% ± 1.17%), and A2 L2/3 (25% shift, 5.06% ± 2.32%; 50% shift, 4.06% ± 1.67%; 75% shift, 8.49% ± 3.09%) respond to both the harmonic sounds and the spectral shifted harmonic stacks. Error bars denote SEM.

To identify if the response of HNs is due to the harmonic relationship between the component sounds rather than the presence of multiple sound components, we performed separate imaging sessions presenting two-tone harmonic stacks and nonharmonic two-tone sounds with the upper-frequency component shifted downward (SHs) by 25, 50 and 75% (Fig. 4*A*). We found that in all three subareas, only <15% of HNs, on average, responded to both the harmonic and its spectrally shifted counterpart (Fig. 4*B*). A one-way ANOVA on each subarea revealed no significant effect of shift levels in the activated proportion of neurons responding to 25, 50, and 75% spectrally shifted harmonic stacks (A1 L2/3, *F*_(2, 16)_ = 0.31; *p* = 0.74; A1 L4, *F*_(2, 16)_ = 1.56; *p* = 0.24; A2 L2/3, *F*_(2, 16)_ = 0.47; *p* = 0.63; Fig. 4*B*). These results suggest that the harmonic relationship between component frequencies, instead of the presence of multiple tones with the same sound level and nonharmonic frequencies, is key to elicit facilitated response of the HNs in all three subareas.

Our findings show that HNs display high sensitivity to harmonic stacks, and the representation of more complex harmonic stacks does not rely on recruiting additional HNs that respond to simpler harmonic stacks. This further supports the hypothesis that ACtx neurons are finely tuned to spectral information within harmonic stacks and represent these features in a sparse and selective manner.

### Nonlinear and linear integration of PT components in harmonically sensitive neurons

**Figure 5.**
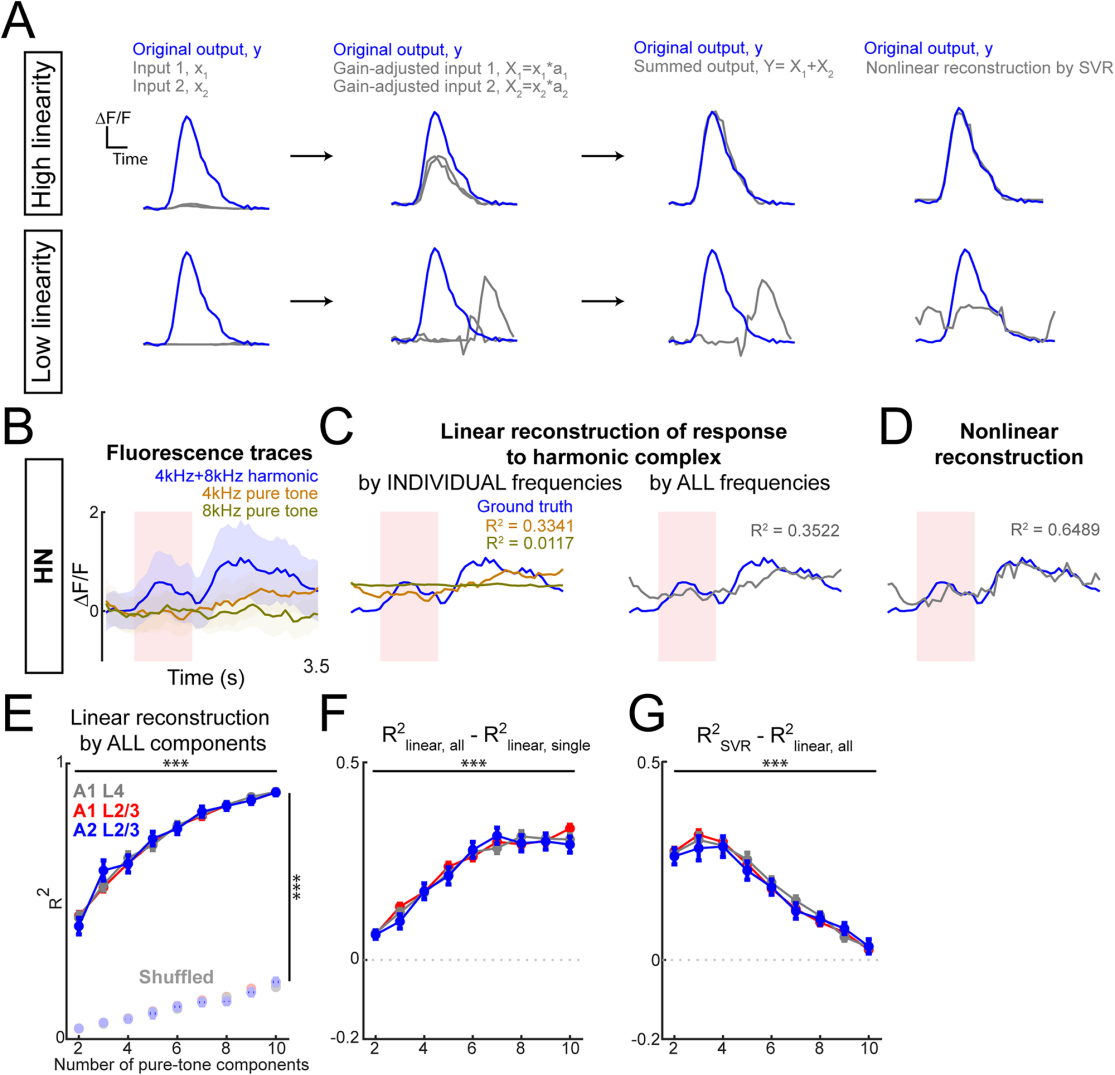
Nonlinearity of response of HNs diminishes when a stack has more harmonic frequencies. ***A***, Schematic showing the linear reconstruction of the simulated output signals by taking the sum of gain-adjusted simulated input signals. Cases of high linearity (top) and low linearity (bottom) are shown. ***B***, Example response to harmonic stacks and PT components of a HN with best harmonic being 4 + 8 kHz stack. the red-shaded area indicates the presence of sound stimuli. The blue-shaded area indicates the standard error of the average response (solid line) to harmonic stacks. Dark yellow (4 kHz) and dark green (8 kHz) shaded areas indicate the SEM of the average response (solid lines). The red-shaded area indicates the presence of sound. ***C***, Linear reconstruction of the example HN response to harmonic by using both the response to the PTs 4 and 8 kHz as variables (left), as well as by the response to each PT separately (right). Higher *R*^2^ tells higher similarity between reconstructed harmonic response by component response and the original response to harmonic. ***D***, Nonlinear reconstruction with SVR that accounts for nonlinearity in the relationship between three responses, which shows higher *R*^2^ score. ***E***, Quantification on the similarity between reconstructed response to harmonic and original response in A1 L4, A1 L2/3, and A2 L2/3. Left, Similarity by using linear reconstruction improves as the harmonic becomes more spectrally complex, which is not shown in permuted response data while the same number of independent variables are used in the reconstruction. ***F***, Improvements on *R*^2^ by using multivariate linear regression compared with univariate linear regression to reconstruct the harmonic response by PT responses. Two-way ANOVA (factors, subareas, harmonic stacks), subareas, *F*_(2, 3,195)_ = 0.7822; *p* = 0.4575; harmonic stacks, *F*_(8, 3,195)_ = 167.9672; *p* = 0; interaction, *F*_(16, 3,195)_ = 0.9670; *p* = 0.4907. The post hoc test revealed significant improvements by using all PT response for linear regression compared with using the best PT response. ***G***, Improvements on *R*^2^ by using nonlinear SVR for reconstruction compared with multivariate linear regression. Two-way ANOVA (factors, subareas, harmonic stacks), subareas, *F*_(2, 3,195)_ = 0.54; *p* = 0.5822; harmonic stacks, *F*_(8,3,195)_ = 111.86; *p* = 0; interaction, *F*_(16, 3,195)_ = 0.43; *p* = 0.9763. The post hoc test revealed diminished improvements on *R*^2^ by nonlinear regression compared with linear multivariate regression as the number of harmonic frequencies becomes larger.

How does the sensitivity to harmonic stacks emerge? Harmonic responses can occur via linear or nonlinear processing of the individual sound components. Given that we identified HN neurons in all areas, these processes might vary between areas. We examined whether the responses of HN neurons to harmonic stacks could be due to processes beyond simple summation of responses to each PT component, suggesting a potential nonlinearity that might vary with increasing spectral complexity of harmonic stimuli. We thus investigated whether HN responses to harmonic stacks were linear or nonlinear functions of the individual PT components. We classified harmonic responses as linear if they could be accurately approximated by a weighted sum of these components as a first-order polynomial function (Fig. 5*A*). Conversely, if the sum could not fully account for the harmonic response, we inferred the presence of nonlinear processing.

To quantify linearity, we first isolated HN responses to their optimal harmonic stimuli and their constituent PT components (Fig. 5*B*). Using these responses, we reconstructed the HN response to harmonic stacks by summing the PT responses through first-order polynomial fitting (Fig. 5*C*). Additionally, we employed SVR to capture nonlinear relationships between harmonic and PT responses (Fig. 5*D*). To assess the linearity, we calculated the *R*^2^ as a measure of the goodness of fit for each polynomial function. To control for the number of predictors in the multivariate function's effect on *R*^2^, we permuted the values of frames of each averaged neuronal response and conducted the same linear reconstruction, allowing us to perform a three-way ANOVA to examine *R*^2^ across three main factors: number of harmonic frequencies, cortical subareas (A1 L4, A1 L2/3, and A2 L2/3), and data type (original vs permuted; Fig. 5*E*). Permutation here allows us to establish a baseline for *R*^2^ performance that accounts for the increasing number of predictors used in our reconstruction model. The three-way ANOVA revealed significant effects of number of harmonic frequencies (*F*_(8, 6,406)_ = 372.44; *p* = 0), data type (*F*_(1, 6,406)_ = 24,375.9; *p* = 0), but no significant effect of subareas (*F*_(2, 6,406)_ = 0.0058; *p* = 0.9942). The interaction between subareas and the number of harmonic frequencies was found to be significant (*F*_(8, 6,406)_ = 147.0647; *p* = 5.8806 × 10^−28^). The post hoc test on the data group (original vs permuted) revealed significantly higher *R*^2^ in the original data group (*p* = 0), indicating the reconstruction using original average sound response achieves better outcomes compared with the average sound response with permuted frames. This result suggests that the temporal structure of fluorescence changes of HNs responding to PTs, despite being too minimal to be considered significant response, contributes to their significant sound-evoked response to harmonic stacks. Such linearity is significantly higher in more complex harmonic stacks while the two-tone stacks are highly nonlinear. In addition, such increase of linearity as the number of harmonic frequencies increase is similarly presented in A1 L2/3, A1 L4, and A2 L2/3. We further examined whether this linear reconstruction was driven by responses to one dominant frequency component or multiple harmonic frequencies components by calculating the differences of *R*^2^ values between single-component and all-component reconstructions across harmonic stacks with the varied number of frequencies (Fig. 5*F*). For more complex harmonic stacks, all-component reconstructions showed significantly higher *R*^2^ values compared with the stacks with fewer frequencies (Fig. 5*F*), implying that the response integration for complex harmonic stacks leverages the contributions of multiple frequency components more linearly.

To probe further into the nonlinear aspects of harmonic processing, we applied SVR to account for nonlinearities in HN responses. We observed improved *R*^2^ with SVR relative to linear reconstruction. However, the degree of improvement diminished as harmonic stacks became more complex (Fig. 5*G*), supporting our finding that HNs responding to more complex harmonic stacks exhibit greater linearity. This heightened linearity in complex harmonic stacks may be due to reduced inhibitory modulation in HNs, while nonlinearity might stem from two primary sources: (1) threshold effects due to intrinsic properties (e.g., ion channel density, leaky conductance, and inhibitory inputs; Gerstner et al., 2014) and (2) amplification effects from positive feedback mechanisms that exponentially boost the PT response into a harmonic response (Wu and Zenke, 2021). In conclusion, our data suggest that HNs processing complex harmonic stacks might experience reduced inhibition and positive feedback compared with those processing spectrally simpler harmonic stacks (Fig. 5*E*–*G*).

### Parallel pathways contribute to the response properties and nonlinear integration in ACtx neurons

ACtx receives parallel ascending inputs from the lemniscal and nonlemniscal pathways (Hackett et al., 2011; Saldeitis et al., 2014; Liu et al., 2019). These pathways shape the functional properties of ACtx neurons, with lemniscal input from the ventral medial geniculate body (MGBv) preferentially driving onset responses (Aitkin and Webster, 1972; Imig and Morel, 1983; Redies and Brandner, 1991; Hackett et al., 2011), while nonlemniscal input from the dorsal medial geniculate body (MGBd) is thought to preferentially contribute to offset responses (He, 2001; Liu et al., 2019). We investigated whether the temporal response properties of the three neuron types—HNs, PTNs, and BNs—aligned predominantly with lemniscal or nonlemniscal input patterns, focusing on their responses to sound onset and offset.

**Figure 6.**
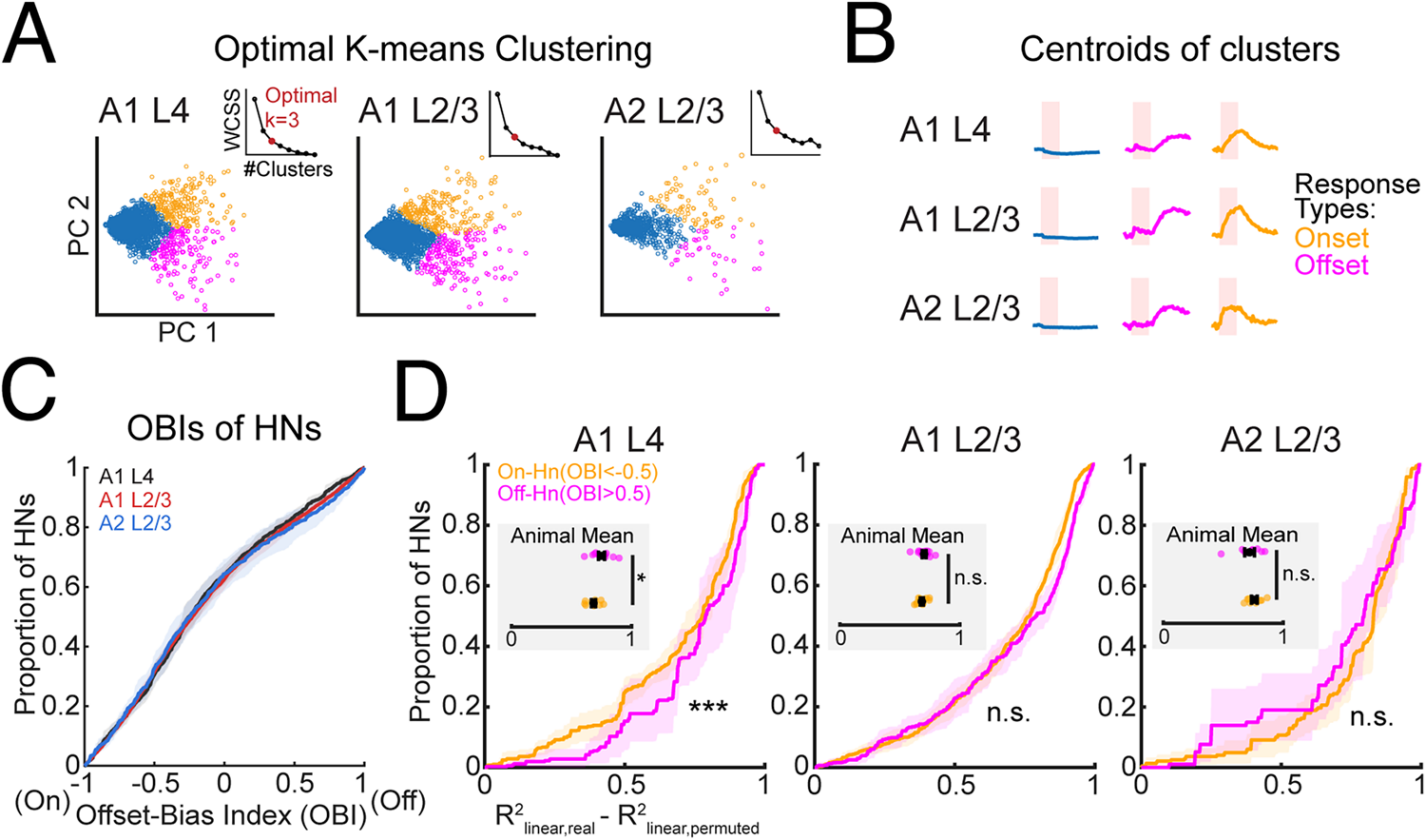
Neurons with offset bias in A1 L4 have more linear integration. ***A***, *K*-means clustering results with optimized number of clusters (*k* = 5) plotted as different colors, denoting five clusters, for three subareas in the top two components from the principle component analysis. Clustering was performed on the average sound-evoked response of HNs to all harmonic stacks, regardless of the responsiveness to each sound. The number of clusters is chosen using the elbow method for each subarea, denoted as the red dot in the dotted line graphs. In A1 L4, *n* = 2,141 average traces from 1,565 neurons. In A1 L2/3, *n* = 4,811 traces from 3,167 neurons. In A2 L2/3, *n* = 1,002 average traces from 631 neurons. ***B***, Centroids of five *k*-means clusters including distinct offset response (magenta) and onset response (orange). ***C***, Cumulative distribution of OBIs of HNs in A1 L4 (gray), A1 L2/3 (red), and A2 L2/3 (blue). On the *x*-axis, −1 denotes total onset biased; 1 denotes total offset biased. *Y*-axis shows the cumulative proportions of the OBIs. Solid lines denote average cumulative distribution curves across animals for each subarea. Shaded areas denote 95% CI of the mean distribution curves. Two-sample Kolmogorov–Smirnov test is performed with post hoc Bonferroni’s correction. Reported *p* values are corrected. A1 L4 versus A1 L2/3, *p* = 1; A1 L4 versus A2 L2/3, *p* = 0.814; A1 L2/3 versus A2 L2/3, *p* = 1. Cumulative distributions of OBIs of PTNs and BNs are available in Extended Data Figure 6-1. The quantification of OBIs of HNs responding to harmonic stacks of varied numbers of frequencies is available in Extended Data Figure 6-2 and the statistical result is available in Extended Data Figure 6-3. ***D***, Cumulative distribution of *R*^2^ of HNs with high bias toward onset (OBI < −0.5, orange) or offset (OBI > 0.5, magenta) by multivariate linear reconstruction of harmonic response in A1 L4, A1 L2/3, and A2 L2/3. Colored solid lines denote the average cumulative distribution curves across animals for A1 L4 (left), A1 L2/3 (middle), and A2 L2/3 (right). The embedded scatterplots show the mean of OBIs of each animal. Two-sample *t* test on the animal means of OBIs for off-HN and on-HN plotted in the cumulated curves: A1 L4, *R*^2^_OBI < −0.5_ = 0.68 ± 0.02; *R*^2^_OBI > 0.5_ = 0.75 ± 0.03; *p* = 0.0426; A1 L2/3, *R*^2^_OBI < −0.5_ = 0.68 ± 0.01; *R*^2^_OBI > 0.5_ = 0.70 ± 0.02; *p* = 0.3539; A2 L2/3; *R*^2^_OBI < −0.5_ = 0.76 ± 0.02; *R*^2^_OBI > 0.5_ = 0.72 ± 0.04; *p* = 0.4033.

To characterize these temporal properties, we applied *K*-means clustering (Fig. 6*A*), revealing distinct clusters dominated by onset or offset responses (Fig. 6*B*). We further quantified each neuron's onset–offset bias using the OBI [OBI = (offset response − onset response) / (offset response + onset response); Liu et al., 2019] and compared OBIs across subareas. A linear mixed-effect model showed that there was no main effect of subarea on the OBIs of HNs (Fig. 6*C*; *F*_(2, 100,090)_ = 0.117; *p* = 0.890), BNs (*F*_(2, 403,880)_ = 0.37912; *p* = 0.6845), and PTNs (*F*_(2, 79,888)_ = 1.3764; *p* = 0.2525). We next examined OBIs of HNs to harmonic stacks of varied complexity and found that the average OBIs of HNs responding to a varied number of harmonic frequencies in three subareas were highly similar (Extended Data Figs. 6-2, 6-3). In summary, all sound-responsive neurons show homogeneous OBIs among the three subareas.

10.1523/ENEURO.0038-26.2026.f6-1 Figure 6-1 **OBIs of PTNs and BNs did not differ among subareas** **Left**: Distribution of OBIs of PTNs of individual subjects in A1 L4 (gray), A1 L2/3 (Red), A2 L2/3 (blue). One-way ANOVA on the main factor of subareas with the use of linear mixed-effect models: F(2,79888) = 1.3764, *p* *=* 0.2525. **Right**: Distribution of OBIs of BNs of individual subjects in A1 L4 (gray), A1 L2/3 (Red), A2 L2/3 (blue). One-way ANOVA on the main factor of subareas with the use of linear mixed-effect models: F(2,403880) = 0.37912, *p* *=* 0.68447. Download Figure 6-1, TIF file.

10.1523/ENEURO.0038-26.2026.f6-2 Figure 6-2 **Average OBIs of HNs responding to varied number of harmonic frequencies are highly similar.** Average OBIs of HNs responding to harmonic stacks with varied number of frequencies were plotted for three subareas. Two-way ANOVA was conducted on the main factors of subareas and harmonic frequencies number. F_subareas_(2,6144) = 0.7297, *p*_subareas_ = 0.482. F_frequencies_(8,6144) = 1.9556, *p*_subareas_ = 0.0480. F_interaction_(16,6144) = 0.9752, *p*_interaction_ = 0.4810. Post-hoc test on the main factor of number of harmonic frequencies did not reveal any significant difference between OBIs of HNs responding to different harmonic conditions (See Supplementary Table 3). Download Figure 6-2, TIF file.

10.1523/ENEURO.0038-26.2026.f6-3 Figure 6-3 **Statistics of average OBIs of HNs responding to varied harmonic stacks.** Report of statistics of post-hoc comparisons between s number of harmonic frequencies following two-way ANOVA on main factors of subareas and number of harmonic frequencies. Download Figure 6-3, DOCX file.

HNs exhibit nonlinear responses to simpler harmonic stacks, consistent with underlying mechanisms such as thresholding, where responses are only elicited when the summed input from multiple components exceeds a certain activation threshold, and rectification, where only positive or suprathreshold inputs drive significantly increased spiking activity, resulting in the increase of fluorescence change from the baseline in this study. Thus, to explore whether these nonlinear dynamics vary in onset or offset neurons, we analyzed the linearity of HNs with different OBIs. Specifically, we selected HNs with higher biases toward onset (On-HNs, OBI < −0.5) or offset (Off-HNs, OBI > 0.5) and examined their response linearity, respectively (Fig. 6*D*). One-way ANOVA with linear mixed-effect models indicates that offset-biased HNs in A1 L4 exhibit a more linear response profile compared with onset-biased HNs (*F*_(1, 4,321)_ = 5.6729; *p* = 0.0173). Off-HNs and on-HNs did not show a significant difference of linearity index in A1 L2/3 (*F*_(1, 10,594)_ = 0.79324; *p* = 0.37314) or A2 L2/3 (*F*_(1, 1,510)_ = 0.62739; *p* = 0.4284). These findings suggest that, while HNs are similarly distributed across cortical subregions, their response dynamics vary, with A1 L4 off-HNs showing a more linear spectral integration. This linearity may result from lower inhibitory modulation and minimal positive feedback during spectral integration in A1 L4. Overall, this variation in response profiles across subregions highlights region-specific processing differences, potentially supporting a distinct role of A1 L4 in spectral integration.

### Robust encoding of harmonic stacks by coactivated harmonic neurons in A1 L4

**Figure 7.**
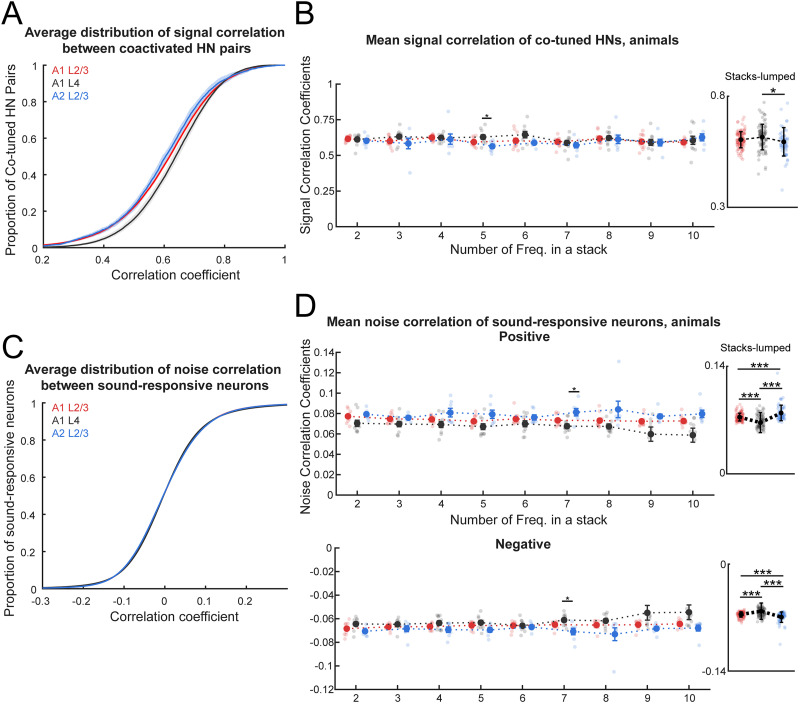
Robust encoding of harmonic stacks by sparse neuron network in A1 L4. ***A***, Distribution of signal correlation coefficients in three subareas. Colored solid lines denote the average cumulated distribution curves of A1 L4 (gray), A1 L2/3 (red), and A2 L2/3 (blue). Colored-shaded areas denote the 95% confidence intervals (CI). The two-sample Kolmogorov–Smirnov test with adjusted *p* values, A1 L4 versus A1 L2/3, *p* = 0.0012; A2 L2/3 versus A1 L2/3, *p* = 0.519; A1 L4 versus A1 L2/3, *p* = 0.102. ***B***, Average signal correlation coefficients (left) of pair-wise signal correlation between coactivated HNs responding to the activating harmonic stacks with varied numbers of components in A1 L4 (gray), A1 L2/3 (red), and A2 L2/3 (blue), as well as scatterplot (right) that shows the stacks-lumped signal correlation coefficients for each subarea. Each data point represents the mean coefficients to one unique harmonic stack of one animal subject. Two-way ANOVA (factors, subareas, number of harmonic frequencies) on the left plot: subareas, *F*_(2, 233)_ = 3.3990; *p* = 0.0351; harmonics, *F*_(8, 233)_ = 1.5078; *p* = 0.1552; interactions, *F*_(16, 233)_ = 0.8349; *p* = 0.6451. The post hoc test on subareas: A1 L4 versus A1 L2/3, *p* = 0.2279; A1 L4 versus A2 L2/3, *p* = 0.0280; A1 L2/3 versus A2 L2/3, *p* = 0.4656. Error bars indicate SEM. Post hoc statistics data are available in Extended Data Figure 7-1. ***C***, Distribution of noise correlation coefficients in three subareas. Colored solid lines denote the average cumulated distribution curves of A1 L4 (gray), A1 L2/3 (red), and A2 L2/3 (blue). Colored-shaded areas denote the 95% CI. The two-sample Kolmogorov–Smirnov test with adjusted *p* values, A1 L4 versus A1 L2/3, *p* = 0.9826; A2 L2/3 versus A1 L2/3, *p* = 0.5111; A1 L4 versus A1 L2/3, *p* = 0.9004. ***D***, Average correlation coefficients of positive (left) and negative (bottom) pair-wise noise correlation of sound-responsive neuron pairs during the presence of harmonic stacks with varied numbers of components in A1 L4 (gray), A1 L2/3 (red), and A2 L2/3 (blue), as well as scatterplots (right) that shows the stack-lumped positive (top) and negative (bottom) noise correlation coefficients for each subarea. Each data point represents the mean coefficients to one unique harmonic stack of one animal subject. Two-way ANOVA on positive coefficients (factors, subareas, number of harmonic frequencies), subareas, *F*_(2, 232)_ = 30.3275; *p* = 1.99 × 10^−12^; harmonics, *F*_(8, 232)_ = 1.1879; *p* = 0.3071; interactions, *F*_(16, 232)_ = 0.827; *p* = 0.6542. The post hoc test on subareas, A1 L4 versus A1 L2/3, *p* = 1.20 × 10^−4^; A1 L4 versus A2 L2/3, *p* = 0; A1 L2/3 versus A2 L2/3, *p* = 3.54 × 10^−5^. Two-way ANOVA on negative coefficients (factors, subareas, number of harmonic frequencies), subareas, *F*_(2, 232)_ = 38.5751; *p* = 3.44 × 10^−15^; harmonics, *F*_(8, 232)_ = 0.633; *p* = 0.7497; interactions, *F*_(16, 232)_ = 0.5479; *p* = 0.919. The post hoc test on subareas, A1 L4 versus A1 L2/3, *p* = 6.57 × 10^−7^; A1 L4 versus A2 L2/3, *p* = 0; A1 L2/3 versus A2 L2/3, *p* = 3.58 × 10^−5^. Post hoc statistics data are available in Extended Data Figure 7-2.

Building upon our investigation of individual HN responses, we next aimed to characterize the collective activity of neurons sensitive to harmonic stacks but not to their PT components. While the proportion of HNs was similar across the three subareas (Fig. 4), prior widefield imaging studies revealed distinct activation patterns for tones and vocalizations in these regions (Calhoun et al., 2023). These differences may stem from varying reliability in how sound stimuli engage neuronal networks across subareas. The sound-evoked response comprises a stimulus-driven component representing the overall response to the stimulus and a variable component that reflects the network's activation pattern. The contributions of these components can be separated by calculating signal and noise correlations: signal correlations capture shared stimulus-driven responses, while noise correlations reflect FC between neurons (Averbeck et al., 2006; Averbeck and Lee, 2006; Cohen and Kohn, 2011; Winkowski and Kanold, 2013; Hazon et al., 2022). We computed the signal correlation between HNs coactivated by each harmonic sound and compared across harmonic complexity and imaged subregions. Our results examined by a two-way ANOVA with the use of linear mixed-effect models showed that changing the number of harmonic frequencies as a main factor did not significantly affect the signal correlation coefficients among HNs (Fig. 7*B*; *F*_(8, 233)_ = 1.51; *p* = 0.1552). Thus, more spectrally complex harmonic processing does not rely on recruiting additional neurons (Fig. 4*D*) nor increasing synchronization of coactivated HNs as the harmonic becomes more spectrally broad (Fig. 7*B*). However, the main effect of subareas is significant (Fig. 7*B*; *F*_(2, 233)_ = 3.4; *p* = 0.0351) with nonsignificant interaction between two main factors (*F*_(16, 233)_ = 0.83; *p* = 0.6451). We followed up with the post hoc test on the significant main factor of subareas (Extended Data Fig. 7-1) and found that HNs in A1 L4 showed significantly higher signal correlation than HNs in A2 L2/3 (*p*_adjusted_ = 0.029). Thus, HNs in A1 L4 respond to harmonic stacks more synchronously than HNs in A2 L2/3. This high degree of coordination in A1 L4 may reflect a specialization for processing spectrally complex sounds, such as harmonic stacks, at an early stage of auditory processing.

10.1523/ENEURO.0038-26.2026.f7-1 Figure 7-1 **Statistics of signal correlations of co-tuned HNs** Statistical report of average signal correlation coefficients across animals for each subarea and each harmonic sound. Download Figure 7-1, DOCX file.

10.1523/ENEURO.0038-26.2026.f7-2 Figure 7-2 **Statistics of positive and negative noise correlation of sound-evoked neurons** Statistical report of average positive and negative correlation coefficients across animals for each subarea and each harmonic sound. Download Figure 7-2, DOCX file.

### Sparse neuronal network inferred by weakest pair-wise noise correlations in A1 L4

Noise correlations capture how fluctuations in neuronal activity, independent of stimulus-evoked response, are shared between pairs of neurons. High noise correlation indicates a high probability of shared connectivity between neurons, and thus noise correlation can serve as a proxy for measuring FC between neuron pairs. To further investigate network dynamics in harmonic processing, we examined whether neurons responding to different stimulus types in the three auditory subareas share higher or lower functional connections. We computed noise correlations between every sound-responsive neuron using the mean-subtracted trial response to harmonic stacks and found highly similar noise correlation between pairs of sound-responsive neurons among the auditory subareas (Fig. 7*C*; one-way ANOVA with linear mixed-effect models, *F*_(2, 903,400)_ = 0.4581; *p* = 0.6325). We then examined the average positive and negative noise correlations for individual mice in three subareas. A two-way ANOVA was conducted to examine the effects of the number of frequencies in a harmonic stack and subareas on the average positive and negative noise correlation coefficients, plotted separately. For noise correlation, there was a main effect of subareas (positive correlation, *F*_(2, 233)_ = 30.33; *p* = 1.99 × 10^−12^; negative correlation, *F*_(2, 233)_ = 38.58; *p* = 3.44 × 10^−15^). However, there was no main effect of number of frequencies for the average positive (*F*_(8, 233)_ = 1.19; *p* = 0.3071) and negative correlations (*F*_(8, 233)_ = 0.63; *p* = 0.7497). There was no interaction between the two factors for the average negative (*F*_(16, 233)_ = 0.55; *p* = 0.919) nor positive correlations (*F*_(16, 233)_ = 0.827; *p* = 0.6542). Post hoc comparisons were performed for subareas (Extended Data Fig. 7-2) and revealed that neuron pairs in A1 L4 showed the weakest noise correlation (i.e., lower positive noise correlation and higher negative correlation) compared with A1 L2/3 (*p*_adjusted, positive_ = 1.2 × 10^−4^; *p*_adjusted, negative_ = 6.47 × 10^−7^) and A2 L2/3 (*p*_adjusted, positive_ = 0; *p*_adjusted, negative_ = 0). A2 L2/3 has the strongest noise correlations compared with A1 L2/3 (*p*_adjusted, positive_ = 3.54 × 10^−5^; *p*_adjusted, negative_ = 3.58 × 10^−5^).

Together, our analysis of noise correlations among neuron types across subareas suggests that sound-responsive neurons in A1 L4 are generally more likely to receive distinct functional inputs, forming sparser, more selective networks than those in L2/3, which exhibit stronger noise correlations, indicating more shared synaptic inputs. This network sparsity in A1 L4 remained consistent despite increasing spectral complexity of harmonic stacks, supporting the idea that the ACtx encodes sounds sparsely and selectively adapts to varying spectral complexity.

### A1 L4 shows the highest BN–HN directed FC

**Figure 8.**
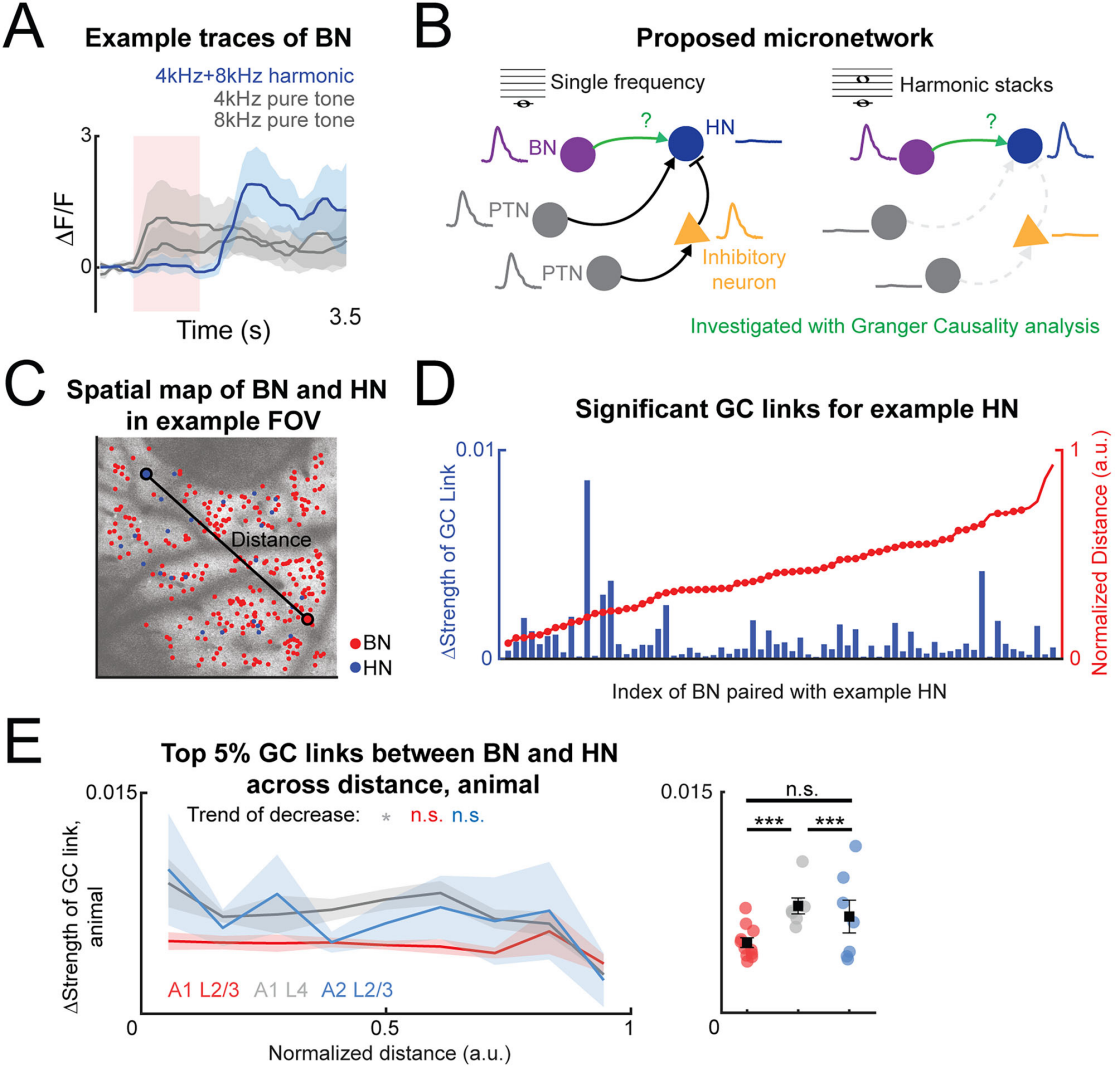
A proposed micronetwork for shaping harmonic-sensitive response of HNs in the auditory pathway. ***A***, Example average sound-evoked responses of BNs to two PTs (black trace) and the corresponding harmonic sound (blue trace). The red-shaded area denotes the presence of sound stimuli. Blue-shaded and gray-shaded areas denotes the standard error of the average response to harmonic stacks and PTs, respectively. ***B***, Schematic showing the proposed micronetwork that can encode how HN responses to sounds with different components. Along the auditory pathway, PTN activates INs when PT sound is played, further inhibiting the HN (left). BN directly activates BN without any inhibition from PTN, which triggers the high responses of HN when harmonic is played. We next investigated the FC between BN and HN by applying GC analysis. ***C***, Example field-of-view images showing the spatial location of PTNs, HNs, and BNs in A1 L2/3. Left, PTNs are located heterogeneously across imaged A1 L2/3. The color indicates best frequency of the plotted neuron. Right, BN and HN are plotted on the same field of view. Larger dots indicate an example coactivated pair of HN and BN to one stimulus, in which the BN achieves the highest *R*^2^ reconstructing the response of HN to the stimulus. The black line indicates the distance between the HN and BN. ***D***, ΔStrengths of significant GC links between all BNs within the same FOV (left *Y*-axis, blue) as an example HN plotted with the distances between each BN and the example HN (right *Y*-axis, red). The distances are normalized by the maximum distance among all BN–HN pairs for all HNs for each animal. ***E***, Quantification of top 5% GC links between any BN–HN pair plotted against binned normalized distances in three subareas (left). A1 L2/3, *n* = 11 mice. A1 L4, *n* = 7 mice. A2 L2/3, *n* = 7 mice. Two separate Kruskal–Wallis tests revealed the significant main effect of subareas (*χ*^2^_(2)_ = 35.3033; *p* = 2.16 × 10^−8^) and nonsignificant effect of distances (*χ*^2^_(8)_ = 13.2486; *p* = 0.1036) on the Δstrength of top 5% GC links. Spearman correlation performed on the GC links as the distances increase reveals significant trend of decrease of GC links (left panel) in A1 L2/3 (*ρ* = −0.2170; *p* = 0.0388) and nonsignificance in A1 L4 (*ρ* = −0.1202; *p* = 0.3731) and A2 L2/3 (*ρ* = −0.2132; *p* = 0.1254). Post hoc Dunn's test with Dunn–Sidak correction showed that A1 L4 has the highest top5% Δstrengths of significant GC links of BN–HN pairs compared with A1 L2/3 (*p* = 1.03 × 10^−8^) and A2 L2/3 (*p* = 5.71 × 10^−4^; right panel).

While multiple network topologies can exist to explain the harmonic-sensitive response of HNs, we devised a simple micronetwork that could explain how a harmonic-sensitive response might be constructed. Given the responsiveness of BNs in both the PT condition and harmonic sound condition (Fig. 8*A*) and their potentially constant signal outputs to HN, BNs might play an additional critical role in enabling the selective response to harmonic stacks in HNs (Fig. 8*B*). As we imaged excitatory neurons, we hypothesized that inhibition could suppress the HN response to PTs. Thus, an HN would receive excitatory drive from a BN as well as input from an inhibitory neuron (IN) driven via a PTN. We thus investigated the plausibility of this simple micronetwork scheme by measuring the directed FC from BN to HN by applying GC analysis on the sound-evoked response of BNs and HNs. We aimed to answer two questions: Do BN–HN have significant FC, and does the FC differ between subareas and across distances?

To answer these questions, we performed the GC analysis on every pair of BN–HN to explore their FC. We compare the FC to the FC in surrogate data, which was constructed by shuffling frames of the whole recording session for individual sound-responsive neurons. We used ΔGC link strength as a metric to quantify the relative strength of GC links. We calculated ΔGC link strength by subtracting any significant absolute GC link values of the surrogate data from the absolute values of the real data. ΔGC link strength was positive, indicating that neurons show nonrandom FC. We next investigated whether ΔGC link strength depended on subarea and distance between the target HN and the BNs in the FOV (Fig. 8*C*). For example, one HN might share higher ΔGC link strength with BNs in shorter distances (Fig. 8*D*). However, from analyzing all BN–HN pairs within FOVs, we found no significant effect of distances on the strength of GC links. On the other hand, the test revealed a significant effect of subareas (Fig. 8*E*). Post hoc tests further showed that BN–HN in A1 L4 had the strongest GC links, followed by A1 L2/3 and A2 L2/3. This result suggests that the BN–HN connectivity in the proposed micronetwork might be more well-represented in A1 L4 compared with the upper cortical layers.

Noise correlations in ACtx decrease with distance (Winkowski and Kanold, 2013) suggesting neurons closer to each other are more likely to receive shared synaptic inputs. Thus, we explored the potential trend of decreased FC as the distances between BN and HN increases by performing Spearman correlation analysis. As an example, we computed the ΔGC link strength from every BN within the same FOV to one example HN and ranked the ΔGC link strength by the distances between BNs and the example HN (Fig. 8*C*,*D*). To avoid bias of the size of subareas, we normalized the distance by dividing the BN–HN distance by the longest distance between sound-responsive neurons within the FOV. We then answered the question of whether ΔGC link strength might decrease as the normalized distance increases between BN and HN by plotting the top 5% values of all BN–HN pairs falling into the binned normalized distances. While A1 L2/3 and A2 L2/3 showed no significant trend as the distances changed, A1 L4 showed a small but significant decrease of the mean values of top 5% GC links as the distance increased (Fig. 8*C*). Such a result suggests that, being different from BNs in A1 L4, BNs in L2/3 do not preferably transfer information only to HNs in its neighborhood but also coordinate with HNs that are farther in the FOV.

Together, we showed that BNs can have significant but small directed excitatory influence onto HNs which is stronger in A1 L4 and weaker in L2/3 of A1 and A2. Specifically, the relatively stronger FC from BN to HN compared with randomized data suggests that the linear inputs from BNs can serve as essential and meaningful building blocks for the nonlinear integration performed by HNs, ultimately shaping their sensitive responses to harmonic stacks. Such BN–HN connectivity in our proposed micronetwork model is better represented in A1 L4. However, due to the small values of GC links observed in BN–HN pairs in ACtx, it is highly likely that HN in ACtx receives inputs from BNs present in the upstream of the auditory pathway, such as MGB or even the inferior colliculus.

## Discussion

We investigated the population encoding of spectrally simple and complex harmonic stacks in different auditory cortical subfields. We find HNs that respond to harmonic stacks but not to individual frequencies. We find that multiple subareas, including A1 L4, A1 L2/3, and A2 L2/3, in the ACtx contain HNs and that the fraction of HNs is similar across A1 and A2. HNs show sensitivity to particular stacks of harmonic frequencies and are characterized by nonlinear integration of the component frequencies. Thus, harmonic sensitivity is already present in A1 L4 and is not a unique feature of any specific auditory cortical subarea but seems to be created independently in multiple subareas.

Simple sounds, such as PTs or harmonic stacks of few frequencies, serve as the fundamental building blocks of more intricate stimuli, such as harmonic stacks of more than five frequencies found in speech. Previous studies have examined the neural representation of specific sound features in the ACtx of humans, rats, and mice (Lewis et al., 2009; Okada et al., 2010; Carruthers et al., 2015; de Heer et al., 2017; O'Sullivan et al., 2019; Staib and Fruhholz, 2023), and intrinsic imaging has suggested that mouse A2 L2/3 is preferentially activated by harmonics (Kline et al., 2021). In contrast, we found that the proportion of neurons activated by harmonic stacks was only slightly higher in A2 L2/3 compared with A1 L2/3 and A1 L4, regardless of number of harmonic frequencies in the stack or the sex of the mice. Moreover, the average amplitude of neuronal responses in A1 L2/3 and A2 L2/3 were similar and higher than A1 L4. The differences between our study and the prior study (Kline et al., 2021) likely lie in the imaging specificity, duration of sounds, as well as the mouse lines used. Instead of intrinsic imaging with low spatial resolution, we used in vivo two-photon imaging and electrophysiology with single-cell resolution. Instead of short duration sounds (100–300 ms), we utilized sound with longer duration (1,000 ms). Certain proportions of neurons may be sensitive to the duration of the sound (Theunissen et al., 2000; Buonomano and Maass, 2009) or require longer stimuli to trigger the significant changes in calcium traces. The prior study utilized C57Bl/6 mice and did not separate animals by sex. Thus, the observed differences could be due to C57Bl/6 mice having early-onset high–frequency hearing loss (Ison et al., 2007; Jendrichovsky et al., 2024) or due to sex-dependent age–related changes in hearing (Shilling-Scrivo et al., 2021, 2022). In contrast, we here use mice that retain good high-frequency hearing across age. Moreover, given the behavioral importance of natural stimuli containing harmonic stacks, early sensory experience could shape the responses in ACtx; thus differences in the rearing environment (Chang and Merzenich, 2003; Sanes and Bao, 2009; Homma et al., 2020; Chang and Kanold, 2021) could underlie the observed differences.

Among HNs in three imaged auditory subfields, we showed that frequency shifts disrupted their response to the two-tone harmonic stacks. In this study, the spectral shift was applied only downward, disrupting harmonicity and narrowing the spectral bandwidth of the stimulus. As a result, the diminished responses across three shift levels (25, 50, and 75%) could reflect sensitivity to either decreased bandwidth or the altered frequency relationship. However, because the response at the 25% shift did not differ significantly from the 75% shift, which assimilates the fundamental frequency that failed to elicit a response in HN, it is unlikely that decreased bandwidth alone explains the reduction. Furthermore, the fact that this reduction was consistent across region with varying average bandwidths suggests that these HNs respond preferentially to specific frequency combinations with the harmonic structure of a two-tone sound. Future studies incorporating upward or bidirectional shifts could be utilized to distinguish the relative contributions of harmonicity and spectral bandwidth.

The harmonic-sensitive response of HNs can be explained by their nonlinear integration of individual frequencies. The integration of harmonic frequencies becomes more linear for HNs responding to stacks with more harmonic components, suggesting that broader frequency integration is associated with greater linearity. Consistent with studies reporting supralinear and sublinear integration in A1 L2/3 and A2 L2/3 for harmonic representation (Kline et al., 2023), our results provided insights of how the linearity of spectral integration can change depending on the spectral contents. Since nonlinear integration was present in A1 L4, it might also exist in the auditory thalamus or earlier in the auditory pathway that already shapes and refines the representations of complex sounds (Patterson et al., 2002; Nelken, 2008; Bartlett, 2013). In all imaged subfields the nonlinearity in spectral integration diminished as the number of harmonic components increased. This is potentially due to altered dynamics of excitatory/inhibitory inputs in the local network. It is also possible that the representation of simpler and more complex harmonic stacks employed the linear and nonlinear pathways, emerging from the cochlear nucleus (Yu and Young, 2000), at different levels depending on the spectral contents of the sounds. These findings underscore the dynamic nature of spectral integration in the ACtx, adapting to the varied complexity of auditory stimuli.

To explain the nonlinear spectral integration of frequencies by HNs, we proposed a micronetwork model in which broadly tuned neurons (BNs) were critical for establishing harmonic-sensitive tuning: they display lower sensitivity for particular harmonic stacks, and their PT response reconstructs harmonic responses with higher linearity. We applied GC analysis as in our prior work (Francis et al., 2018; Sheikhattar et al., 2018) and revealed a small but significantly stronger directed influence from BNs to HNs in A1 L4 compared with L2/3 of A1 and A2. This result suggests that the underlying network may differ between subregions, and we speculate that the BN→HN connectivity exists in or spans multiple regions and enables the tuning profiles of HNs in the ACtx. Future experiments with higher temporal resolution such as electrophysiology or in vivo optogenetic single-cell manipulation (Kang et al., 2025) could provide strong evidence to support this proposed network.

To explore the sources of nonlinearity, we examined whether nonlinear neurons might show preference toward the sound onset or offset, which are essential for auditory scene analysis (Bregman, 1994). HNs, BNs, and PTNs in the three subareas were similar in their onset/offset bias. Notably only in A1 L4, offset-biased HNs tended to be more linear, while onset-biased HNs were more nonlinear. Thus, the onset pathway may encode spectrally complex sounds more linearly, whereas the nonlemniscal offset pathway shows increased nonlinearity. Thus, neurons in the thalamocortical recipient layer showing distinct onset or offset bias might receive more distinct lemniscal and nonlemniscal inputs compared with L2/3.

Coactivated HNs in A1 L4 exhibit similar average signal correlations as A1 L2/3 but higher correlations compared with those in A2 L2/3, indicating that neurons in L2/3 may exhibit a more distributed representation of harmonic features, resulting in lower sound-evoked synchronization than in A1 L4. Thus, A1 L4 may play a foundational role in the initial encoding of harmonic structure, while L2/3 may contribute to higher-order processing of sound features. The neuronal network involving all sound-evoked neurons in A1 L4 was the sparsest, showing the weakest average noise correlation, compared with A1 L2/3 and A2 L2/3, whereas A2 L2/3 neurons showed strongest average noise correlations. This observation represents the hierarchical transformation of neural processing from A1 L4 to A2 L2/3, suggesting that neurons in the thalamocortical recipient layer have fewer shared synaptic inputs despite stronger stimulus encoding than in higher-order areas, where neurons in A2 L2/3 showed significantly more FC. This result is consistent with PT-responsive neurons in A1 L4 showing higher signal correlations and lower noise correlations than those in A1 L2/3 (Winkowski and Kanold, 2013).

Altogether, our results show that harmonically sensitive neurons are ubiquitous in A1 and A2, indicating the importance of harmonic integration for auditory processing. Thus, already, A1 L4 functions as a center for integrating spectral information for complex sounds, supporting robust encoding through coordinated but sparsely connected networks.

## Synthesis

Reviewing Editor: David Schoppik, New York University Grossman School of Medicine Decisions are customarily a result of the Reviewing Editor and the peer reviewers coming together and discussing their recommendations until a consensus is reached. When revisions are invited, a fact-based synthesis statement explaining their decision and outlining what is needed to prepare a revision will be listed below. The following reviewer(s) agreed to reveal their identity: NONE. Note: If this manuscript was transferred from JNeurosci and a decision was made to accept the manuscript without peer review, a synthesis may not be available.

I've reviewed the manuscript, past reviews, and authors's responses. I believe the manuscript in its current form is well-suited for eNeuro.
