# Supplementary figures and images for: Robust Representation and Nonlinear Spectral Integration of Harmonic Stacks in Layer 4 of the Mouse Primary Auditory Cortex
Source: eNeuro. 2026 Mar 18;13(3):ENEURO.0038-26.2026. doi: 10.1523/ENEURO.0038-26.2026 (PMC13002317; doi:10.1523/ENEURO.0038-26.2026)

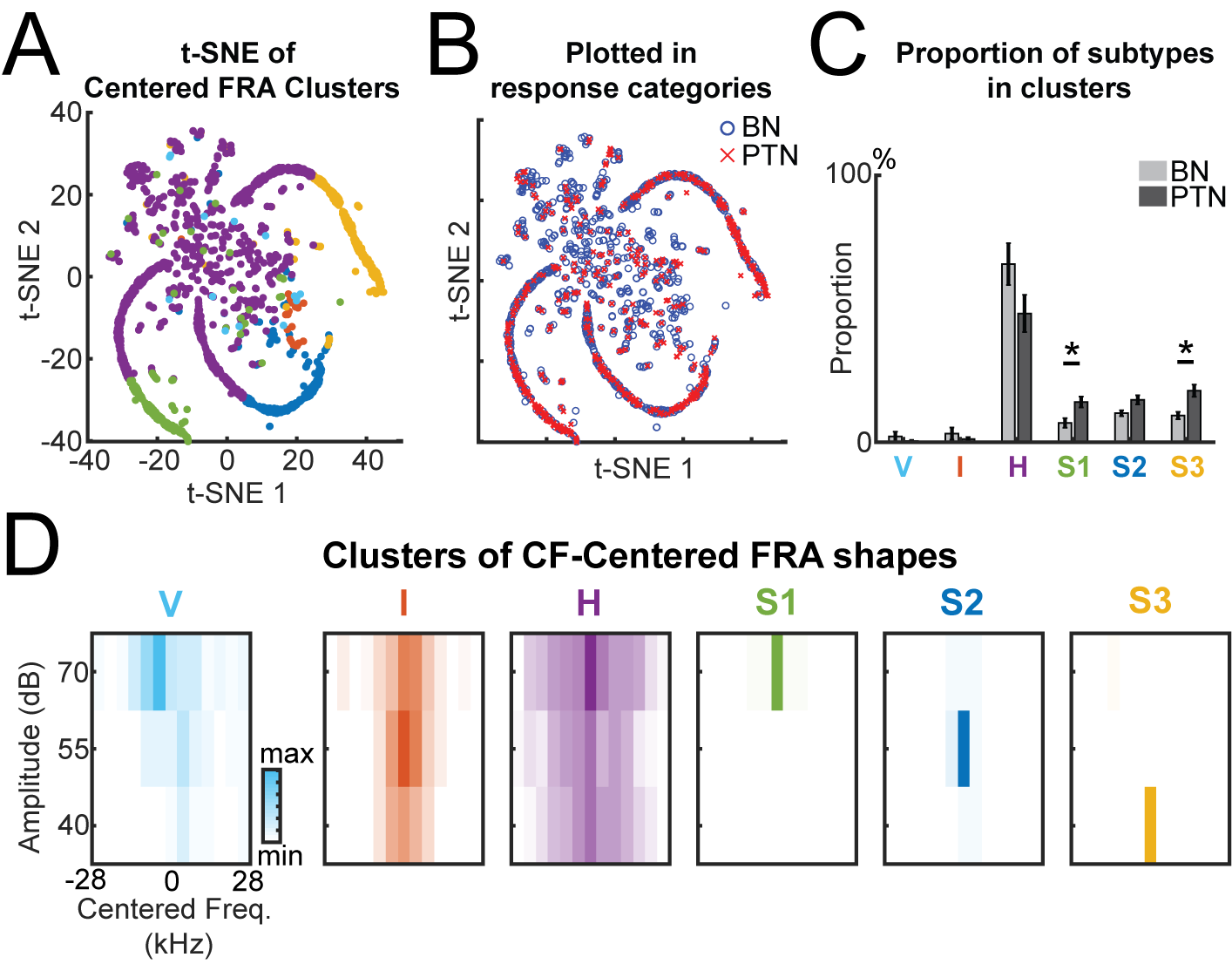

Supplement: Figure 1-1 — Distinct frequency response area (FRA) profiles of both-responding neurons (BNs) and pure-tone neurons (PTNs) in A1 L2/3. A: t-SNE visualization of k-means clustering applied to centered FRA profiles from sound-responsive neurons (n = 3 animals, 1418 neurons). B: Same t-SNE plot as in (A), now colored by BN and PTN identity, illustrating the distribution of neuron subtypes across FRA-based clusters. C: Proportion of BNs and PTNs within each cluster, showing differential cluster occupancy by neuron subtype. Error bars indicates standard errors. *: p < 0.05. Two-sample t-test on proportions of BN versus PTN for each cluster: V, p = 0.4125; I, p = 0.4543; H, p = 0.1531; S1, p = 0.0445; S2, p = 0.0680; S3, p = 0.0249. D: Average centered FRA maps for each of the six clusters, revealing distinct spectral and intensity tuning profiles. Vertical bars indicate peak response frequency. Download Figure 1-1, TIF file. [file eneuro-13-ENEURO.0038-26.2026-s001.tif]

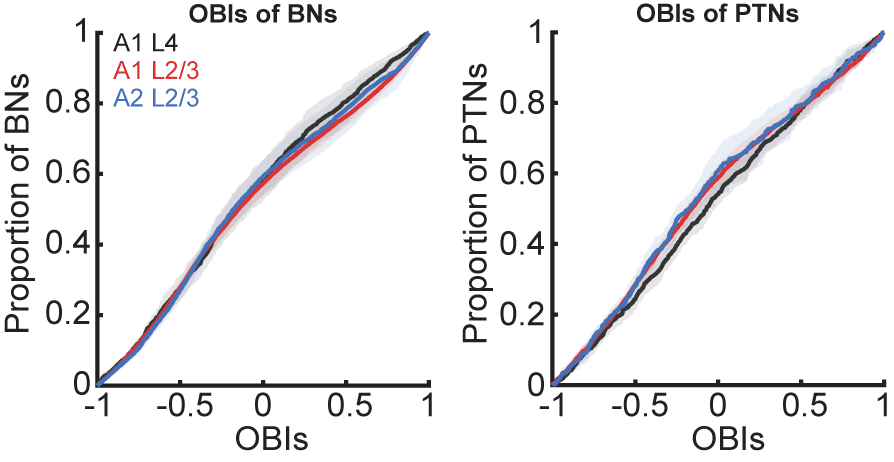

Supplement: Figure 6-1 — OBIs of PTNs and BNs did not differ among subareas Left: Distribution of OBIs of PTNs of individual subjects in A1 L4 (gray), A1 L2/3 (Red), A2 L2/3 (blue). One-way ANOVA on the main factor of subareas with the use of linear mixed-effect models: F(2,79888) = 1.3764, p = 0.2525. Right: Distribution of OBIs of BNs of individual subjects in A1 L4 (gray), A1 L2/3 (Red), A2 L2/3 (blue). One-way ANOVA on the main factor of subareas with the use of linear mixed-effect models: F(2,403880) = 0.37912, p = 0.68447. Download Figure 6-1, TIF file. [file eneuro-13-ENEURO.0038-26.2026-s004.tif]

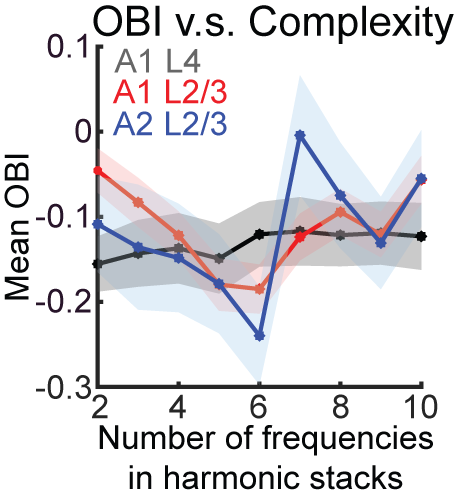

Supplement: Figure 6-2 — Average OBIs of HNs responding to varied number of harmonic frequencies are highly similar. Average OBIs of HNs responding to harmonic stacks with varied number of frequencies were plotted for three subareas. Two-way ANOVA was conducted on the main factors of subareas and harmonic frequencies number. Fsubareas(2,6144) = 0.7297, psubareas = 0.482. Ffrequencies(8,6144) = 1.9556, psubareas = 0.0480. Finteraction(16,6144) = 0.9752, pinteraction = 0.4810. Post-hoc test on the main factor of number of harmonic frequencies did not reveal any significant difference between OBIs of HNs responding to different harmonic conditions (See Supplementary Table 3). Download Figure 6-2, TIF file. [file eneuro-13-ENEURO.0038-26.2026-s005.tif]
