# Supplementary material for: Robust Representation and Nonlinear Spectral Integration of Harmonic Stacks in Layer 4 of the Mouse Primary Auditory Cortex
Source: eNeuro. 2026 Mar 18;13(3):ENEURO.0038-26.2026. doi: 10.1523/ENEURO.0038-26.2026 (PMC13002317; doi:10.1523/ENEURO.0038-26.2026)
Supplement: Figure 3-1 — Statistics of HN proportions activated by varied harmonic stacks Report of statistics of average HN proportion across animals for each subarea and each harmonic sound. Download Figure 3-1, DOCX file. [file eneuro-13-ENEURO.0038-26.2026-s002.docx]

**Extended Data Figure 3-1**

|  | Number of frequencies | Mean (%) | SEM (%) |
| --- | --- | --- | --- |
| A1 L4 | 2 | 10.43 | 2.61 |
|  | 3 | 10.85 | 2.06 |
|  | 4 | 9.93 | 2.48 |
|  | 5 | 9.06 | 2.25 |
|  | 6 | 11.25 | 1.99 |
|  | 7 | 8.59 | 2.4 |
|  | 8 | 9.29 | 2.8 |
|  | 9 | 9.26 | 2.48 |
|  | 10 | 8.91 | 2.57 |
| A1 L2/3 | 2 | 11.99 | 1.69 |
|  | 3 | 11.29 | 1.6 |
|  | 4 | 10.17 | 0.92 |
|  | 5 | 9.09 | 0.98 |
|  | 6 | 10.09 | 1.7 |
|  | 7 | 9.42 | 1.17 |
|  | 8 | 10 | 1.49 |
|  | 9 | 8.92 | 0.94 |
|  | 10 | 9.78 | 1.18 |
| A2 L2/3 | 2 | 13.46 | 2.04 |
|  | 3 | 10.38 | 1.74 |
|  | 4 | 12.37 | 2.2 |
|  | 5 | 10.94 | 1.27 |
|  | 6 | 12.13 | 1.64 |
|  | 7 | 12.18 | 0.87 |
|  | 8 | 12.46 | 1.1 |
|  | 9 | 12.98 | 2.3 |
|  | 10 | 13.36 | 1.52 |
