# Supplementary material for: Robust Representation and Nonlinear Spectral Integration of Harmonic Stacks in Layer 4 of the Mouse Primary Auditory Cortex
Source: eNeuro. 2026 Mar 18;13(3):ENEURO.0038-26.2026. doi: 10.1523/ENEURO.0038-26.2026 (PMC13002317; doi:10.1523/ENEURO.0038-26.2026)
Supplement: Figure 3-2 — Two-sample t-test comparing HN proportions between subareas for each sound Statistical report of pair-wise comparisons across HN proportions activated by harmonic sounds with different numbers of frequencies, following two-way ANOVA on main factors of subareas and number of harmonic frequencies. Download Figure 3-2, DOCX file. [file eneuro-13-ENEURO.0038-26.2026-s003.docx]

**Extended Data Figure 3-2**

| **Bonferroni-corrected p-values of two-sample t-test** | | | | |
| --- | --- | --- | --- | --- |
|  | | Two-sample comparisons between subareas | | |
| Groups | Num. of Frequencies | A1 L2 vs A1 L4 | A1 L2 vs A2 L2 | A1 L4 vs A2 L2 |
| %neurons | 2 | 1 | 1 | 0.5478 |
|  | 3 | 1 | 1 | 1 |
|  | 4 | 1 | 0.345 | 0.8241 |
|  | 5 | 1 | 0.2106 | 0.8895 |
|  | 6 | 1 | 0.5409 | 1 |
|  | 7 | 1 | 0.0261 | 0.1749 |
|  | 8 | 1 | 0.1596 | 0.4317 |
|  | 9 | 1 | 0.0276 | 0.3285 |
|  | 10 | 1 | 0.0183 | 0.1245 |
| %HN | 2  3 | 0.7584  1 | 0.5658  0.4518 | 1  0.9735 |
|  | 4 | 1 | 1 | 1 |
|  | 5 | 0.1926 | 1 | 1 |
|  | 6 | 1 | 1 | 1 |
|  | 7 | 0.2769 | 1 | 1 |
|  | 8 | 0.2046 | 0.5796 | 0.1533 |
|  | 9 | 0.8175 | 1 | 0.4776 |
|  | 10 | 0.261 | 0.453 | 0.0252 |
| %BN | 2 | 0.2457 | 1 | 0.3714 |
|  | 3 | 1 | 1 | 0.9996 |
|  | 4 | 0.5469 | 0.3903 | 0.252 |
|  | 5 | 0.6732 | 1 | 0.498 |
|  | 6 | 1 | 0.3201 | 0.7041 |
|  | 7 | 1 | 0.1155 | 0.2157 |
|  | 8 | 0.765 | 0.1497 | 0.1941 |
|  | 9 | 1 | 0.0639 | 0.1476 |
|  | 10 | 1 | 0.0834 | 0.1794 |
