# Supplementary material for: Robust Representation and Nonlinear Spectral Integration of Harmonic Stacks in Layer 4 of the Mouse Primary Auditory Cortex
Source: eNeuro. 2026 Mar 18;13(3):ENEURO.0038-26.2026. doi: 10.1523/ENEURO.0038-26.2026 (PMC13002317; doi:10.1523/ENEURO.0038-26.2026)
Supplement: Figure 6-3 — Statistics of average OBIs of HNs responding to varied harmonic stacks. Report of statistics of post-hoc comparisons between s number of harmonic frequencies following two-way ANOVA on main factors of subareas and number of harmonic frequencies. Download Figure 6-3, DOCX file. [file eneuro-13-ENEURO.0038-26.2026-s006.docx]

**Extended Data Figure 6-3**

| **Statistics of post-hoc Tukey-Kramer test on the number of harmonic frequencies** | | | | | |
| --- | --- | --- | --- | --- | --- |
| Number of harmonic frequencies | |  | | | |
| Group A | Group B | Lower Limit | Difference | Upper Limit | P-value |
| 2 | 3 | -0.099 | 0.0173 | 0.1336 | 0.9999 |
| 2 | 4 | -0.0802 | 0.0325 | 0.1451 | 0.9933 |
| 2 | 5 | -0.047 | 0.0659 | 0.1787 | 0.6752 |
| 2 | 6 | -0.0313 | 0.0785 | 0.1884 | 0.3941 |
| 2 | 7 | -0.1338 | -0.0216 | 0.0906 | 0.9996 |
| 2 | 8 | -0.1114 | -0.0063 | 0.0987 | 1 |
| 2 | 9 | -0.0845 | 0.0203 | 0.1251 | 0.9996 |
| 2 | 10 | -0.133 | -0.0251 | 0.0827 | 0.9985 |
| 3 | 4 | -0.1069 | 0.0152 | 0.1373 | 1 |
| 3 | 5 | -0.0737 | 0.0486 | 0.1709 | 0.9495 |
| 3 | 6 | -0.0583 | 0.0612 | 0.1808 | 0.8111 |
| 3 | 7 | -0.1606 | -0.0389 | 0.0828 | 0.9867 |
| 3 | 8 | -0.1388 | -0.0236 | 0.0915 | 0.9994 |
| 3 | 9 | -0.1119 | 0.003 | 0.1179 | 1 |
| 3 | 10 | -0.1601 | -0.0424 | 0.0753 | 0.9718 |
| 4 | 5 | -0.0854 | 0.0334 | 0.1523 | 0.9944 |
| 4 | 6 | -0.0699 | 0.0461 | 0.1621 | 0.9496 |
| 4 | 7 | -0.1723 | -0.0541 | 0.0642 | 0.8911 |
| 4 | 8 | -0.1503 | -0.0388 | 0.0727 | 0.9773 |
| 4 | 9 | -0.1234 | -0.0121 | 0.0991 | 1 |
| 4 | 10 | -0.1717 | -0.0576 | 0.0566 | 0.8241 |
| 5 | 6 | -0.1036 | 0.0127 | 0.1289 | 1 |
| 5 | 7 | -0.2059 | -0.0875 | 0.0309 | 0.3472 |
| 5 | 8 | -0.1839 | -0.0722 | 0.0395 | 0.5399 |
| 5 | 9 | -0.157 | -0.0455 | 0.0659 | 0.9407 |
| 5 | 10 | -0.2053 | -0.091 | 0.0233 | 0.2474 |
| 6 | 7 | -0.2157 | -0.1002 | 0.0154 | 0.1521 |
| 6 | 8 | -0.1935 | -0.0849 | 0.0238 | 0.2717 |
| 6 | 9 | -0.1666 | -0.0582 | 0.0502 | 0.7678 |
| 6 | 10 | -0.215 | -0.1036 | 0.0077 | 0.0918 |
| 7 | 8 | -0.0958 | 0.0153 | 0.1263 | 1 |
| 7 | 9 | -0.0688 | 0.042 | 0.1527 | 0.9619 |
| 7 | 10 | -0.1172 | -0.0035 | 0.1102 | 1 |
| 8 | 9 | -0.0769 | 0.0267 | 0.1302 | 0.9969 |
| 8 | 10 | -0.1254 | -0.0188 | 0.0879 | 0.9998 |
| 9 | 10 | -0.1518 | -0.0454 | 0.0609 | 0.9242 |
