# Supplementary material for: Robust Representation and Nonlinear Spectral Integration of Harmonic Stacks in Layer 4 of the Mouse Primary Auditory Cortex
Source: eNeuro. 2026 Mar 18;13(3):ENEURO.0038-26.2026. doi: 10.1523/ENEURO.0038-26.2026 (PMC13002317; doi:10.1523/ENEURO.0038-26.2026)
Supplement: Figure 7-1 — Statistics of signal correlations of co-tuned HNs Statistical report of average signal correlation coefficients across animals for each subarea and each harmonic sound. Download Figure 7-1, DOCX file. [file eneuro-13-ENEURO.0038-26.2026-s007.docx]

**Extended Data Figure 7-1**

| **Average signal correlation coefficients, animals** | | | | |
| --- | --- | --- | --- | --- |
| Subareas | Number of frequencies |  | mean | SEM |
| A1 L2/3 | 2 |  | 0.6159635 | 0.0055809 |
|  | 3 |  | 0.5999762 | 0.0104777 |
|  | 4 |  | 0.6249747 | 0.0056381 |
|  | 5 |  | 0.5936893 | 0.0084964 |
|  | 6 |  | 0.6028683 | 0.0159962 |
|  | 7 |  | 0.5944996 | 0.0093402 |
|  | 8 |  | 0.6175332 | 0.0119776 |
|  | 9 |  | 0.596218 | 0.0140747 |
|  | 10 |  | 0.5925663 | 0.0053282 |
| A1 L4 | 2 |  | 0.6119913 | 0.0130026 |
|  | 3 |  | 0.6319068 | 0.0159019 |
|  | 4 |  | 0.6232023 | 0.0092964 |
|  | 5 |  | 0.6287246 | 0.0161097 |
|  | 6 |  | 0.6455124 | 0.02241 |
|  | 7 |  | 0.5871736 | 0.0171571 |
|  | 8 |  | 0.621769 | 0.0153564 |
|  | 9 |  | 0.5915211 | 0.0206645 |
|  | 10 |  | 0.6056294 | 0.0283606 |
| A2 L2/3 | 2 |  | 0.6024677 | 0.0139595 |
|  | 3 |  | 0.5832657 | 0.0367058 |
|  | 4 |  | 0.6138353 | 0.0360477 |
|  | 5 |  | 0.563668 | 0.0144335 |
|  | 6 |  | 0.5882472 | 0.0091033 |
|  | 7 |  | 0.5710379 | 0.018381 |
|  | 8 |  | 0.6126053 | 0.0292716 |
|  | 9 |  | 0.5899745 | 0.0228911 |
|  | 10 |  | 0.6264383 | 0.0246254 |
