# Supplementary material for: Robust Representation and Nonlinear Spectral Integration of Harmonic Stacks in Layer 4 of the Mouse Primary Auditory Cortex
Source: eNeuro. 2026 Mar 18;13(3):ENEURO.0038-26.2026. doi: 10.1523/ENEURO.0038-26.2026 (PMC13002317; doi:10.1523/ENEURO.0038-26.2026)
Supplement: Figure 7-2 — Statistics of positive and negative noise correlation of sound-evoked neurons Statistical report of average positive and negative correlation coefficients across animals for each subarea and each harmonic sound. Download Figure 7-2, DOCX file. [file eneuro-13-ENEURO.0038-26.2026-s008.docx]

**Extended Data Figure 7-2**

|  |  | Positive noise correlation coefficients | | Negative noise correlation coefficients | |
| --- | --- | --- | --- | --- | --- |
| Subareas | Number of frequencies | mean | SEM | mean | SEM |
| A1 L2/3 | 2 | 0.0772864 | 0.0016343 | -0.0684242 | 0.0013868 |
|  | 3 | 0.0747775 | 0.0011917 | -0.0669071 | 0.0007336 |
|  | 4 | 0.0742815 | 0.0010075 | -0.0666779 | 0.0007512 |
|  | 5 | 0.0724514 | 0.001513 | -0.0656767 | 0.0009014 |
|  | 6 | 0.074722 | 0.0015874 | -0.0659317 | 0.0010045 |
|  | 7 | 0.0733297 | 0.001411 | -0.0650243 | 0.0008492 |
|  | 8 | 0.0730573 | 0.001798 | -0.065348 | 0.0010439 |
|  | 9 | 0.0723732 | 0.00143 | -0.0650869 | 0.0010238 |
|  | 10 | 0.0727395 | 0.0013377 | -0.0644117 | 0.0008352 |
| A1 L4 | 2 | 0.0704397 | 0.0030034 | -0.0644473 | 0.0016606 |
|  | 3 | 0.0696415 | 0.0026 | -0.0647269 | 0.0014192 |
|  | 4 | 0.0692357 | 0.0029169 | -0.0636306 | 0.0018544 |
|  | 5 | 0.0671073 | 0.0028514 | -0.0633027 | 0.001775 |
|  | 6 | 0.0699064 | 0.0029554 | -0.0659469 | 0.0015931 |
|  | 7 | 0.0675919 | 0.0022159 | -0.0611138 | 0.0015684 |
|  | 8 | 0.0673202 | 0.0024039 | -0.0617789 | 0.0017299 |
|  | 9 | 0.0597607 | 0.0069985 | -0.0549503 | 0.0062717 |
|  | 10 | 0.0587539 | 0.006907 | -0.0545186 | 0.0062338 |
| A2 L2/3 | 2 | 0.0792857 | 0.0018004 | -0.0705984 | 0.0014228 |
|  | 3 | 0.0758409 | 0.001244 | -0.0684503 | 0.0025214 |
|  | 4 | 0.0808818 | 0.0042615 | -0.0693365 | 0.0023449 |
|  | 5 | 0.0792546 | 0.0040615 | -0.0695107 | 0.0019138 |
|  | 6 | 0.0761668 | 0.0025694 | -0.0670024 | 0.0013149 |
|  | 7 | 0.0812218 | 0.0035769 | -0.0708668 | 0.00275 |
|  | 8 | 0.0840472 | 0.0080723 | -0.0731356 | 0.0054395 |
|  | 9 | 0.0772228 | 0.0022366 | -0.0683432 | 0.0015001 |
|  | 10 | 0.0796779 | 0.0036282 | -0.0679236 | 0.0025304 |
